# A new p65 isoform that bind the glucocorticoid hormone and is expressed in inflammation liver diseases and COVID-19

**DOI:** 10.1038/s41598-021-02119-z

**Published:** 2021-11-25

**Authors:** Gaetano Spinelli, Giuseppa Biddeci, Anna Artale, Francesca Valentino, Giuseppe Tarantino, Giuseppe Gallo, Fabrizio Gianguzza, Pier Giulio Conaldi, Salvatore Corrao, Francesco Gervasi, Tommaso Silvano Aronica, Aldo Di Leonardo, Giovanni Duro, Francesco Di Blasi

**Affiliations:** 1 Istituto per la Ricerca e l’Innovazione Biomedica del Consiglio Nazionale delle Ricerche, Via Ugo La Malfa 153, 90146 Palermo, Italy; 2 Dipartimento di Medicina Specialistica, Diagnostica e Sperimentale, Università di Bologna, Via Massarenti 9, 40138 Bologna, Italy; 3 Dipartimento Scienze e Tecnologie Biologiche Chimiche e Farmaceutiche, Università degli Studi di Palermo, Viale Delle Scienze, ed. 16, 90128 Palermo, Italy; 4 Dipartimento di Ricerca, IRCCS-ISMETT, Istituto Mediterraneo per i Trapianti e Terapie ad alta Specializzazione, Via Tricomi 5, 90127 Palermo, Italy; 5 Unità COVID, Dipartimento di Medicina Interna, Azienda Ospedaliera di Rilevanza Nazionale e Alta Specializzazione ARNAS Civico, Di Cristina, Benfratelli, 90127 Palermo, Italy; 6 Dipartimento PROMISE, Università degli Studi di Palermo, 90100 Palermo, Italy; 7 Laboratorio Specialistico di Oncologia, Rilevanza Nazionale e Alta Specializzazione Ospedaliera Trust ARNAS Civico, Di Cristina, Benfratelli, 90127 Palermo, Italy; 8 Unità Organizzativa Complessa di Patologia Clinica, Rilevanza Nazionale e Alta Specializzazione ARNAS Civico, Di Cristina, Benfratelli, 90127 Palermo, Italy

**Keywords:** Immunology, Inflammation, Molecular biology, Transcription

## Abstract

Inflammation is a physiological process whose deregulation causes some diseases including cancer. Nuclear Factor kB (NF-kB) is a family of ubiquitous and inducible transcription factors, in which the p65/p50 heterodimer is the most abundant complex, that play critical roles mainly in inflammation. Glucocorticoid Receptor (GR) is a ligand-activated transcription factor and acts as an anti-inflammatory agent and immunosuppressant. Thus, NF-kB and GR are physiological antagonists in the inflammation process. Here we show that in mice and humans there is a spliced variant of p65, named p65 iso5, which binds the corticosteroid hormone dexamethasone amplifying the effect of the glucocorticoid receptor and is expressed in the liver of patients with hepatic cirrhosis and hepatocellular carcinoma (HCC). Furthermore, we have quantified the gene expression level of p65 and p65 iso5 in the PBMC of patients affected by SARS-CoV-2 disease. The results showed that in these patients the p65 and p65 iso5 mRNA levels are higher than in healthy subjects. The ability of p65 iso5 to bind dexamethasone and the regulation of the glucocorticoid (GC) response in the opposite way of the wild type improves our knowledge and understanding of the anti-inflammatory response and identifies it as a new therapeutic target to control inflammation and related diseases.

## Introduction

Nuclear Factor kB (NF-kB) are ubiquitous transcription factors that consist of a family of proteins, that play critical roles in inflammation, immunity, cell proliferation, differentiation, and survival ^1^. In mammals there are five members of the transcription factor NF-kB, these are RelA (p65), RelB, c-Rel and the precursor proteins p105 and p100, that are processed into p50 and p52 respectively^2^. All these members share a conserved long amino-terminal “Rel Homology Domain” (RHD) located in the amino terminus within which there are binding, dimerization, interaction with inhibitors (IkB) and nuclear translocation sequences^3–5^. These proteins exert their functions by binding as homodimers or heterodimers to 9–10 base pair DNA sites called *“kB consensus”*. Different combination of these members is responsible for the regulation of diverse target genes through the ability to recognize various DNA-binding sites with different affinities. This diversity contributes to peculiar physiological responses after distinct stimuli^6^. Under resting conditions, NF-kB dimers are bound to inhibitory IkB proteins that sequester NF-kB complexes in the cytoplasm. After a specific signal, the IkB kinase (IKK) complex induces IkB protein phosphorylation, followed by ubiquitination and degradation that lead to NF-kB translocation to the nucleus to control gene expression^7^. The dysregulation of NF-kB activity is linked to autoimmune and metabolic diseases, inflammatory disorders and cancer^8^. NF-kB plays a pivotal role in the activation of the immune response, modulates cell proliferation and differentiation as well as cell death^9^. Coronavirus disease 2019 (COVID-19) caused by Severe Acute Respiratory Syndrome Corona Virus 2 (SARS-CoV-2) has emerged in China and drastically spread throughout the world quickly and declared by World Health Organization as pandemic. Clinical manifestations of COVID-19 may differ from person to person, primarily are characterized by Acute Respiratory Distress Syndrome (ARDS) but an elevated level of pro-inflammatory molecules has been reported as the key pathogenic hallmark of COVID-19^10,11^. It is also very well known the involvement of NF-kB pathway in the regulation and in the pathogenesis of inflammatory diseases^12,13^. NF-kB and Glucocorticoid Receptor (GR) respectively mediate pro- and anti-inflammatory physiological balance. GR is a ligand-activated transcription factor belonging to the superfamily of nuclear receptors. After binding glucocorticoids, GR translocates to the nucleus regulating target genes expression by recognizing Glucocorticoid Responsive Element (GRE) in specific promoters and acts as anti-inflammatory agent and immunosuppressant^14,15^. GR represses NF-kB activation blocking its access to DNA binding sites (-kB) by a protein–protein interaction. It also induces the synthesis of the NF-kB inhibitor IkB^16^. Thus, NF-kB and GR are physiological antagonists in the inflammation process^17^. It has been shown that NF-kB pathway also plays a major role in the development of liver diseases correlated with inflammation like fibrosis and hepatocellular carcinoma (HCC)^18–20^. Alternative splicing is a physiological process in eukaryotes and allows to produce different mRNAs from a single gene. Splicing is often tightly regulated in a tissue specific manner producing diverse mRNAs encoding multiple proteins with distinct biological effects^21–23^. In this report we describe and characterize a new alternative splicing form of p65, named p65 isoform 5 (p65 iso5) related to human product, that is functionally distinct from the known isoforms of p65.

## Results

### Genomic organization of p65 iso5

**Figure 1:**
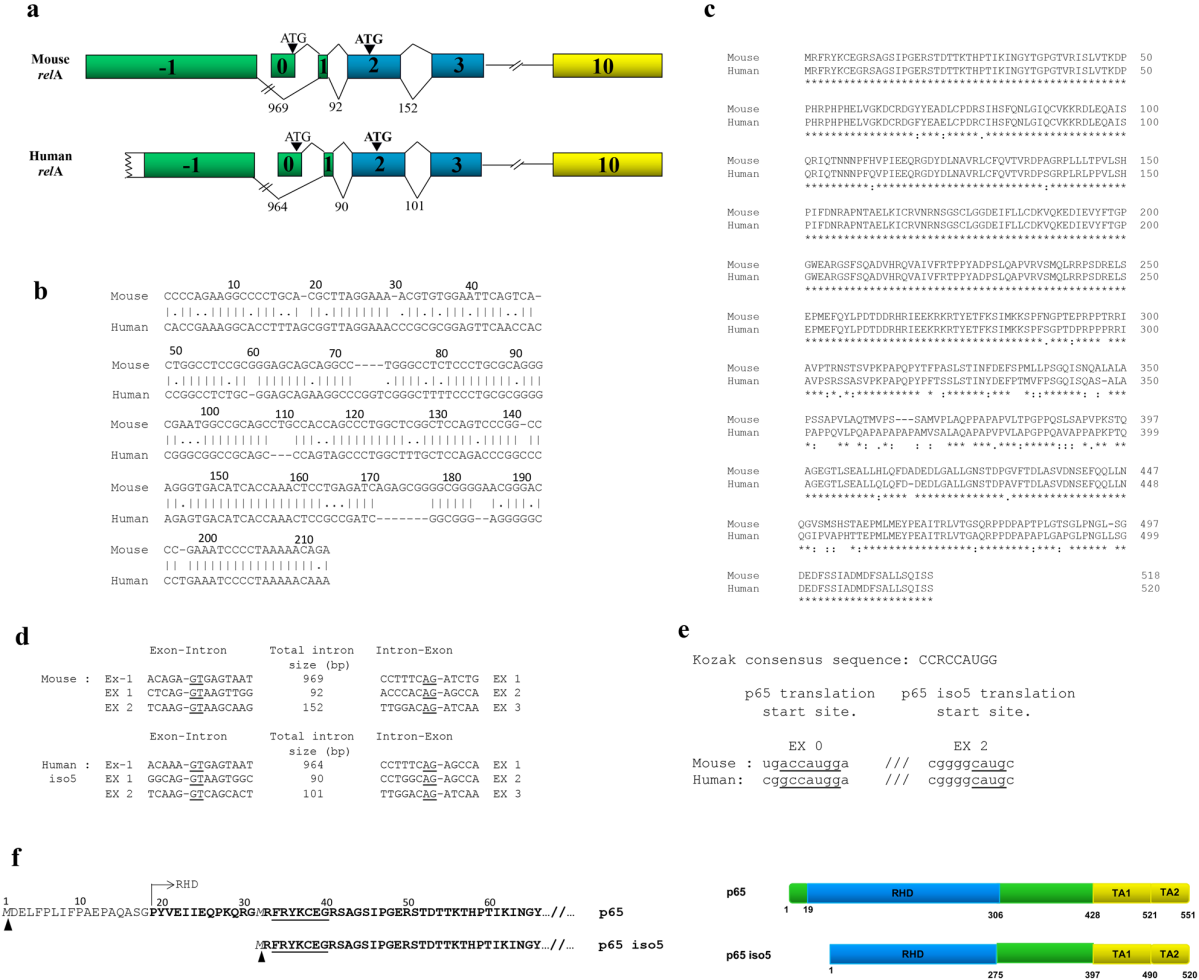
Organization of the murine and human *relA* gene, homology between amino acid sequences of human and mouse isoforms. (**a**) Partial genomic organization of the murine (GenBank accession number MN508964) and human (GenBank accession number MN508965) *relA* gene showing the alternatively spliced exon − 1. The lengths of the introns are indicated in base pairs and p65 iso5 start codon is indicated in bold. (**b**) Comparison of mouse and human exon − 1 partial sequences. (**c**) Alignment between amino acid sequences of p65 iso3 mouse and p65 iso5 human. The alignment was performed by using Clustal Omega tool (EMBL-EBI). (**d**) Exon–intron borders of part of the murine and the human *relA* gene. The intronic splice donor and acceptor sites are underlined. (**e**) Sequences surrounding p65 and p65 iso5 translation start site in mouse and human mRNA. The homology between Kozak consensus sequence of p65 and p65 iso5 are underlined. R indicate a purine (A or G). (**f**) Primary amino acid sequences of human p65 and p65 iso5 proteins. Underlined: NF-κB/Rel domain signature. Bold: Rel Homology Domain starts at amino acid 19. Italics: first methionine for either p65 or p65 iso5.

These investigations started by screening of a mouse cDNA library for the study of differentially expressed p65 binding proteins. Here, a clone with a different *relA* 5′ end gene arrangement has been isolated. In order to verify if this gene corresponded to an alternative spliced form a series of reverse transcriptase and polymerase chain reactions (RT-PCR) were performed on RNA extracts from mouse and human tissues. Using specific oligonucleotides for the exon 2 and oligonucleotides located upstream of the 5′ untranslated region (5′ UTR) of the p65 gene we found an alternative spliced form of p65 mRNA. This new isoform of p65 was named p65 iso5 (Deposited sequence: GenBank accession number MN508965) and contained a previously unknown exon, (named exon − 1) located upstream to the first known exon of p65 (exon 0) (Fig. 1a). A 5′-RACE mapping performed with mouse brain RNA shown that the exon − 1 has a length of 498 base pair (bp) and has a strong homology (73%) with the 213 bp of the exon − 1 that was cloned from human brain RNA (Fig. 1b). The homology between amino acid sequences of human and mouse isoforms is shown (Fig. 1c). Because two splice variants of p65 are already known in mouse, if referred to this organism, this new gene product is called p65 isoform 3 (p65 iso3) (Deposited sequence: GenBank accession number MN508964). p65 iso5 spliced the exon − 1 and exon 1 and skipped exon 0. Consensus intronic sequences at splice junctions were present in the murine and human exon − 1 (Fig. 1d). The exon − 1 for p65 iso5 is transcribed in the same direction of the entire *relA* gene both in mouse and in human and it is a non-coding region. These evidences support the presence of a different promoter region upstream of the previously known. The p65 iso5 ORF (Open Reading Frame) starts at methionine 32 present in the exon 2 (Fig. 1a). This ATG is bordered by a Kozak consensus-like sequence in both the human and the mouse transcripts (Fig. 1e). Consequently, the resulting p65 iso5 protein lacked the amino acid residues 1–31 and had a shorter RHD that contains the dimerization, nuclear-localization and DNA-binding domains (Fig. 1f).

### Identification of p65 iso5 mRNA in human and mouse tissues and the capacity of its protein product to bind kb consensus

**Figure 2:**
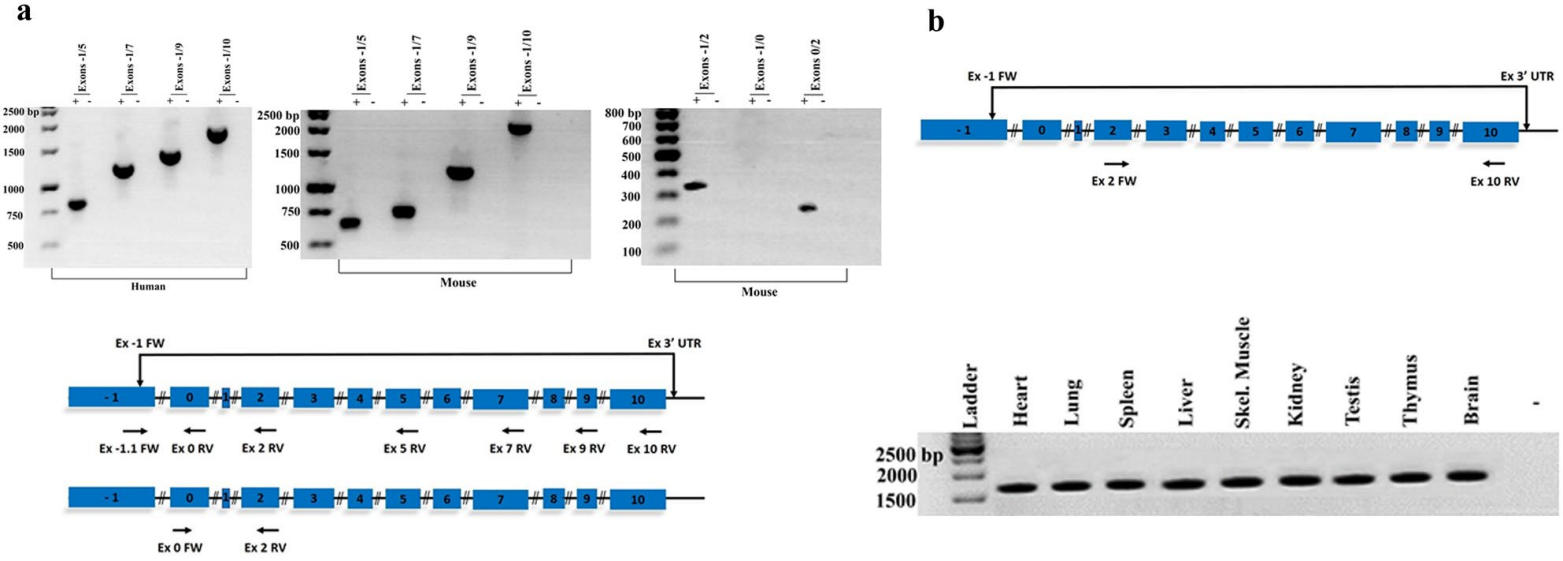
Nested RT-PCR analysis of p65 iso5 expression in RNA samples from human and mouse brain in different mouse tissues. (**a**) First round PCR reaction was performed with specific oligonucleotides for the exon − 1 and 3′UTR (untranslated region). For human and mouse RNA, subsequent RT-PCR reactions were performed with oligonucleotide specific for exon-1 and exon 5; exon 7; exon 9; exon 10 (the entire transcript). PCR analysis of mouse brain RNA were performed with specific primers for: exon − 1 and exon 2; exon − 1 and exon 0; exon 0 and exon 2. The + and − symbols indicate the presence and the absence respectively of reverse transcriptase in the reaction. (**b**) Nested RT-PCR performed on mouse RNA samples extracted from different tissues. First round PCR was performed with primers located on exon − 1 and 3′UTR region, second round PCR was performed with oligonucleotides specific for exon 2 and 10. The − symbol indicates the absence of reverse transcriptase in the reaction (representative of each sample). The full length images of the gels are available in the “Supplementary Information” file.

**Figure 3:**
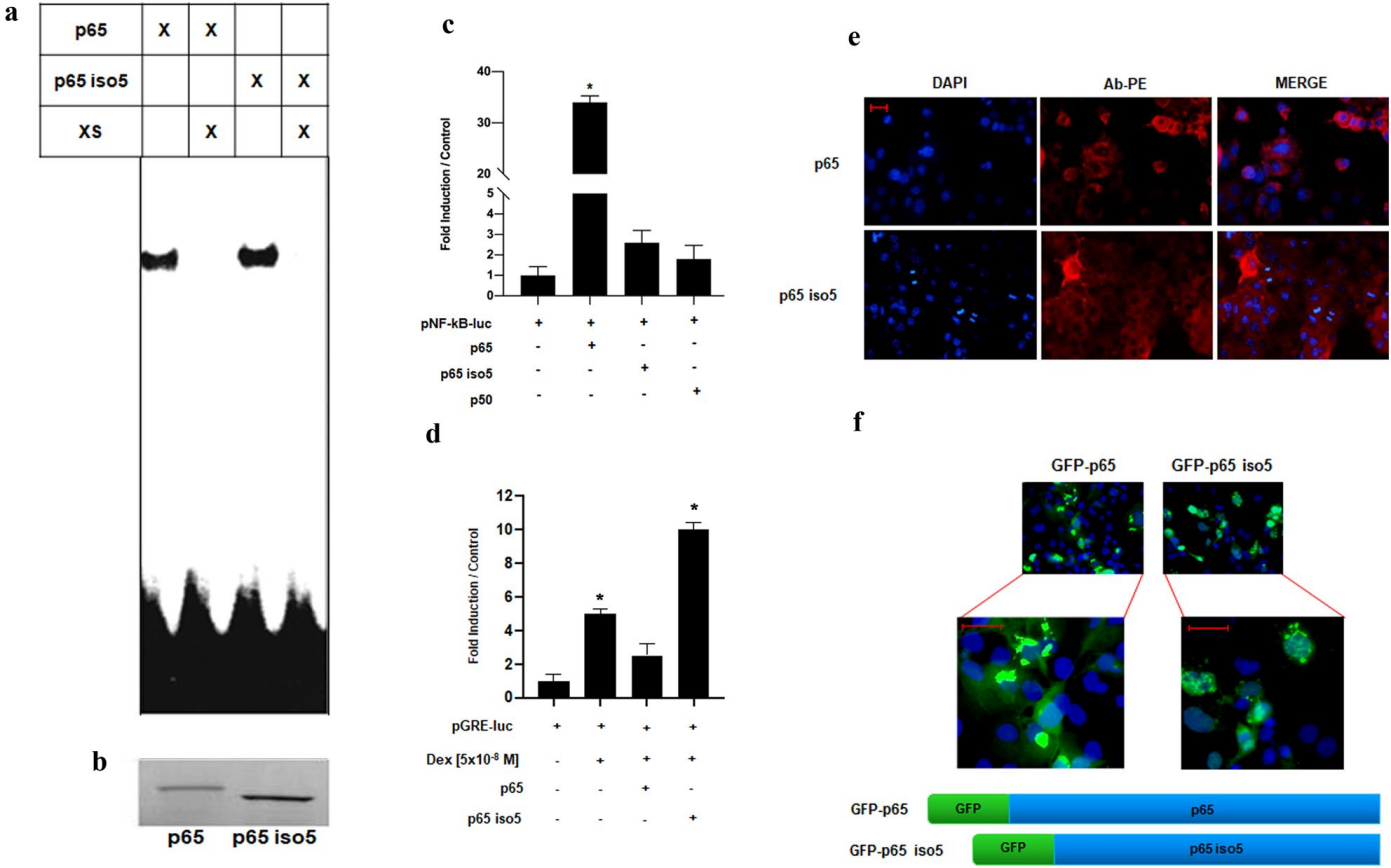
Biochemical and functional analysis of p65 iso5 protein and localization in the cell. (**a**) Electro mobility shift assay (EMSA) showing p65 and p65 iso5 binding activity to the radiolabelled NF-κB consensus sequence. The specific bands were suppressed by 100-fold excess of unlabelled probe. The full length image of the gel is available in the “Supplementary Information” file. (**b**) Polyacrylamide gel electrophoresis showing the in vitro transcribed and translated radiolabelled p65 and p65 iso5 proteins. The membrane has been cut prior the exposition. (**c**) Analysis of the transcriptional activity of p65 and p65 iso5. HeLa cells were transfected with a luciferase reporter driven by consensus NF-κB response elements and with the indicated expression vector. (**d**) Opposite effects of p65 iso5 and p65 on the transcriptional activity of the glucocorticoid receptor (GR) induced by dexamethasone. HeLa cells were transfected with luciferase reporter driven by specific glucocorticoid response elements (GRE) and the indicated plasmids. After transfection, GR was activated by the administration of the glucocorticoid agonist dexamethasone. (**e**) Representative of immunofluorescence of Cos-1 cells transfected with p65 and p65 iso5. In red we identified the protein using NF-κB p65 (L8F6) PE conjugate antibody that recognizes C-terminal proteins, nuclei are evidenced in blue. (**f**) Localization of p65 and p65 iso5 fused at 5′ with GFP (green fluorescent protein), nuclei are evidenced in blue. Data information: In (**b**, **c**), data are presented as mean ± SEM. *P < 0.001, in comparison to cells transfected with reporter plasmid. (GRE) and the indicated plasmids. After transfection, GR was activated by the administration of the glucocorticoid agonist dexamethasone. The full length images of EMSA and of the in vitro transcribed and translated radiolabelled p65 and p65 iso5 proteins are available in the “Supplementary Information” file.

Using nested PCR with primers specific for exon − 1 and exon 5–10, the entire transcripts containing the − 1 exon were found both in mouse and human brains RNA (Fig. 2a). p65 iso5 appears as an alternative splice variant of the p65 gene and no transcript containing both the − 1 and the 0 exons were found when PCR was performed with a set of specific oligos for exons − 1/2 and no transcript were observed using specific oligos for exons − 1/0 (Fig. 2a). Entire p65 iso5 mRNA was present in all the nine mouse tissues we analyzed by nested PCR (Fig. 2b) and in human liver or peripheral blood mononuclear cells (PBMC). Both p65 isoforms were able to bind NF-kB consensus DNA as shown by an Electro Mobility Shift Assay (EMSA). For this experiment, expression plasmids encoding p65 iso5 and p65 wild type (wt) were in vitro transcribed and translated and the final product used for EMSA, using a radiolabelled oligonucleotide containing the NF-kB binding motif (Fig. 3a,b). The p65 iso5 mRNA was detected in every tissue we examined and both p65 proteins are able to bind to DNA.

### Transcriptional activity of p65 iso5 on NF-kB and GRE promoters and cell localization

Despite p65 iso5 is able to bind to DNA, it activates transcription trough canonical NF-kB responsive elements ten time less than p65 (Fig. 3c). These results indicate that in addition to the two already known trans-activating domains mapped within the COOH terminal region of p65^24^, the small portion deleted in the NH_2_ terminal region of the RHD domain of p65 iso5 is also involved in conferring transcriptional activity to p65. NF-kB and GR are oppositely regulated and repress each other transcriptional activity^25,26^. Considering that the physical interaction between p65 and GR has been reported, the domains required for a direct interaction between the two proteins are also present in the p65 iso5 protein^27^. Administration of the selective GR agonist dexamethasone activated the GRE-dependent reporter as expected and this effect was largely reduced by p65. Strikingly, cotransfection with p65 iso5 had exactly the opposite effect doubling dexamethasone-induced gene transcription (Fig. 3d). To determine if the p65 iso5 ability to doubling dexamethasone effect is GR dependent, we performed a luciferase assay in Cos-1 cells that do not produce any steroid hormone receptors. The results showed that in the absence of GR there is no amplification on the GRE reporter gene with p65 iso5, indicating that the effect of p65 iso5 with dexamethasone is GR dependent (Supplementary Fig. S1). Moreover, through immunofluorescence analysis, we demonstrate a different localization of p65 iso5 and p65. The p65 iso5 is primarily localized in the nucleus and in the perinuclear region while p65 is distributed in the cytoplasm (Fig. 3e,f).

### Transcriptional activity of p65 iso5 on human interleukin 6 and TNFα promoters

**Figure 4:**
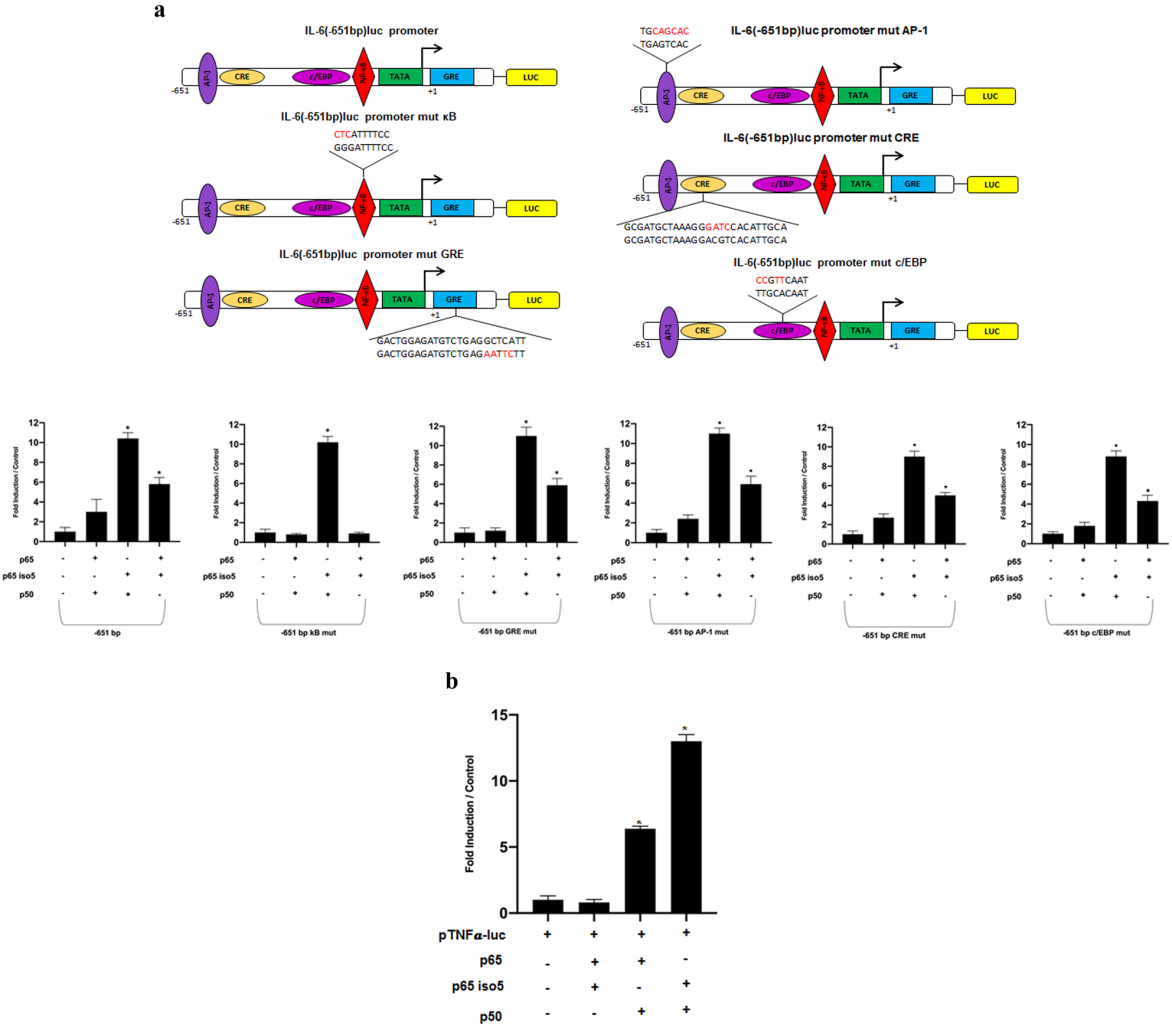
Transcriptional regulation of p65 iso5 on human IL-6-luc and TNFα-luc promoters. (**a**) Effect of p65 iso5 on natural human promoter IL-6-luc. HeLa cells were cotransfected with a luciferase reporter driven by promoter IL6-651luc; IL6-651luc mut κB; IL6-651luc mut GRE; IL-6-651luc mut AP-1; IL-6-651luc mut CRE; IL-6-651luc mut c/EBP and the plasmids p50, p65 and p65 iso5. (**b**) HeLa cells were cotransfected with a luciferase reporter driven by promoter TNFα-luc and the indicated plasmids. Cells were maintained in RPMI 1640 supplemented with Charcoal–dextran (CD) Fetal Bovine Serum (FBS). Data information: in (**a**, **b**), data are presented as mean ± SEM. *P < 0.001, in comparison to cells transfected with reporter plasmid.

Because it is common knowledge that NF-kB is a dimer among the members of Rel structural-related proteins that regulates several genes involved in inflammation, we analyzed the p65 iso5 transcriptional activity with either p50 or p65 wt on human interleukin 6 (IL-6) or tumor necrosis factor-α (TNFα) promoters. IL-6 is a cytokine that is produced during inflammation playing a key role in the acute phase response process. In addition, IL-6 can also drive chronic inflammation^28,29^. To investigate whether p65 iso5 may have an influence on IL-6 expression, we transfected HeLa cells with pIL6-luc651 promoter construct^30^ with heterodimers combination of the three members p50, p65, p65 iso5. As shown in Fig. 4a the p65 iso5/p50 and the p65 iso5/p65 activate transcription more efficiently than the classical p65/p50 dimer. Mutation in the consensus of AP-1, or CRE, or C/EBP binding sites did not significantly affect the transactivation activity of every dimer except for the classical p65/p50 dimers on AP-1 mutated consensus binding sites (Fig. 4a). Surprisingly, despite point mutation of the NF-kB consensus sequence abolished the activation in both dimers containing the p65 wt, the trans-activating capacity of p65 iso5/p50 was unaffected at all suggesting a different way to regulate this promoter region. A clear reduction of trans-activating activity of the dimer containing the p65 iso5/p50 was detected in the mutant on the GRE sequence (Fig. 4a). TNFα is a multifunctional cytokine produced by several types of cells in particular cells of the monocytic lineage^31,32^ playing a key role in the regulation of immune cells. Therefore, we studied the possible effects of p65 iso5 protein with either p50 or p65 on human TNFα promoter with or without dexamethasone. The results demonstrate that the heterodimer p65 iso5/p50 has a greater trans-activating activity compared to the classic heterodimer p65/p50 in presence of dexamethasone^33^ (Fig. 4b). The data obtained on IL-6 and TNFα promoters shows a different regulation capacity of p65 iso5 compared to p65 wt. The ability of this new isoform to activate the promoters of some target genes, in a different way compared to wild type, suggests that p65 iso5 could help providing different biochemical properties to NF-kB depending on the partners involved in the complex.

### Hypothetical binding of p65 iso5 and dexamethasone

**Figure 5:**
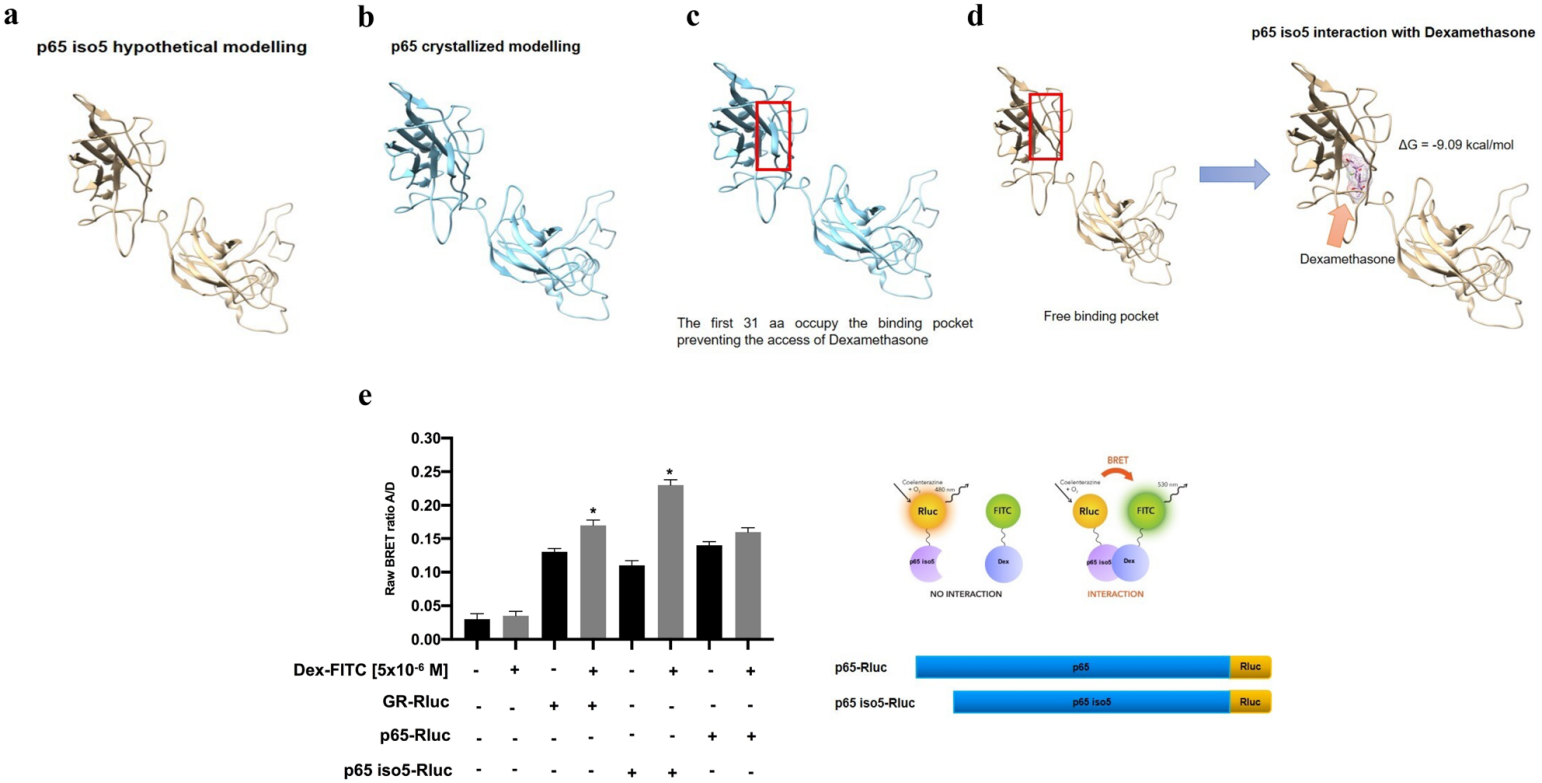
Modelling and dexamethasone binding of p65 iso5. (**a**) The p65 iso5 ipotetical modelling was determined with Modeller v9.8. (**b**) The p65 crystallized structure obtained from the complex IκBα/NF-κB (PDB: 1NFI). (**c**, **d**) The docking simulations of the p65 and p65 iso5 were performed with Autodock v4.2 program. In the red box is highlighted the β-sheet element of the first 31 amino acids of the wild-type protein while in the p65 iso5 the 31 amino acids are missing and create a free pocket for the dexamethasone binding. **(e**) The Bioluminescence Resonance Energy Transfer (BRET) assay shows the in vivo interaction between the p65 iso5 and dexamethasone. Cos-1 cells were cotransfected with the BRET constructs and treated with Dex-FITC, the raw BRET ratio was calculated by the A/D (λ 530 nm acceptor/λ 495 nm donor). Data information: in (**f**), data are presented as mean ± SEM. *P < 0.001, in comparison to cells transfected without Dex-FITC.

Since a possible reason of the ability of p65 iso5 to doubling dexamethasone effect could be determined by a direct binding of p65 iso5 with dexamethasone, we have performed in silico docking simulations in order to investigate the potential of the dexamethasone to bind the p65 iso5 protein. Since the crystallized structure of the complex IkBα/NF-kB was available (PDB: 1NFI) and the p65 iso5 differs from the wt by the mere lack of the first 31 residues, a protein structure model of the p65 iso5 was created with Modeller v9.8^34–36^ as shown in on Fig. 5a,b. In addition, the Autodock v4.2 program^37^ was used to understand if also p65 has the potential to bind the dexamethasone (Supplementary Fig. S2). However, the simulations did not find any docking clusters on the p65 wt. The docking analysis between the p65 iso5 model and the dexamethasone revealed four different docking clusters and the top scoring docked model was selected (Supplementary Fig. S2). The model was characterized by a binding energy of ΔG (kcal/mol) = − 9.09 a value comparable to the one of the binding dexamethasone-human GR ΔG (kcal/mol) = − 11.57 (Supplementary Movie S1). The docking simulation suggests that dexamethasone targets a binding pocket that was occupied by the first 31 amino acids in the wt protein (Fig. 5c). These 31 aa, that are missing in the p65 iso5 (Fig. 5d), create a β-sheet element that does not allow the binding of the dexamethasone in the wt structure (Supplementary Movie S2).

### In vitro binding interaction of p65 iso5 protein with dexamethasone

In order to have an experimental demonstration of the interaction between p65 iso5 and dexamethasone, the Bioluminescence Resonance Energy Transfer (BRET) technique^38^ has been performed. We used the Renilla luciferase (Rluc) as the donor and Dexamethasone Fluorescein (Dex-FITC) as the acceptor. We prepared the full-length GR, p65 and p65 iso5 constructs with Rluc fused in frame with the C-terminal regions (Fig. 5e). We initially examined the GR-Rluc/Dex-FITC interaction as control and all the experiments were performed on Cos-1 cells because this cell line does not produce any steroid hormone receptors. As shown on Fig. 5e, since GR binds the Dex the raw BRET ratio increases in the presence of Dex-FITC. We used the p65-Rluc construct as negative control and no differences of raw BRET signal ratio were observed with and without Dex-FITC. Finally, an increase of raw BRET ratio, similar to GR-Rluc/Dex-FITC, was observed when p65 iso5-Rluc construct was used. These results confirm that p65 iso5 bind the dexamethasone. So far, GR is the only known receptor able to mediate actions of glucocorticoids on gene transcription and regulation. The capacity of p65 iso5 to bind dexamethasone and the regulation of the glucocorticoid responses in the opposite way of the wild type, opens a new paradigm that needs to be clarified with regard to human inflammation-related diseases.

### Evaluation of the mRNA expression levels of p65 and p65 iso5 in PBMC from COVID-19 patients

**Figure 6:**
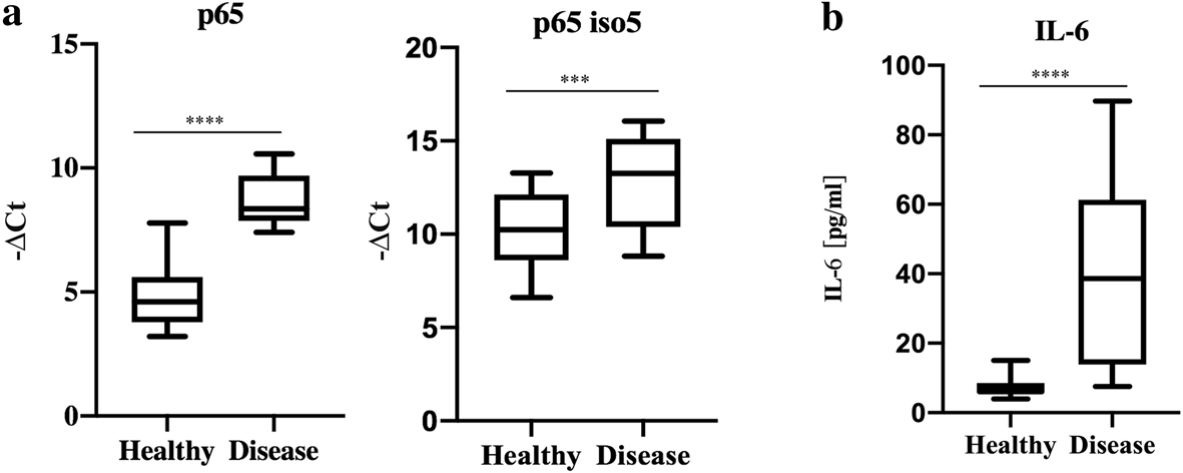
mRNA relative quantity of p65 and p65 iso5 and level IL-6 in blood. (**a**) The Ct and median measuring gene expression levels of p65 and p65 iso5 in PBMC of SARS-CoV-2 and in control patients. The p65 and p65 iso5 mRNA are significantly up-regulated in patients with SARS-CoV-2 compared to control samples. (**b**) IL-6 levels and median measured in patients with SARS-CoV-2 compared to healthy patients. In (**a**, **b**) data are presented as mean ± SEM. ****< 0.001 and ***< 0.005 in comparison to control patients.

As it has been pointed out in the study conducted by Huang et al.^39^ the levels of TNF-α, IL-2, IL-10, IL-7, and other inflammatory markers were higher in patients suffering from COVID-19 than in healthy subjects. Moreover, it has been observed that the most severe cases of COVID-19 are associated with a reduction in CD4^+^ T, T-lymphocytes, CD8^+^ T cells and an increase in levels of C-reactive protein, D-dimer, ferritin, IL-6, IL-2R^40^. Moreover, the transcription factor NF-kB regulates the activity of the immune cells and is involved in the regulation of cytokines gene expression. Taking this into account, an abnormal activation of the NF-kB activity could be associated with cytokine storm and multiple organ defects in SARS-CoV-2 infection^41^. Furthermore, immunomodulation at the level of NF-kB activation and the inhibition of IkB degradation along with TNF-α inhibition will potentially result in a reduction in the cytokine storm and alleviate the severity of COVID-19. Taking into consideration the data obtained on IL-6 and TNFα promoters, in which p65 iso5 shows a different regulation compared with p65 wt, we decided to study gene expression profiles by qPCR, for p65 and p65 iso5 in patients affected by SARS-CoV-2 infection. As shown in Fig. 6a, we found that the expression levels of p65 and p65 iso5 mRNA are up-regulated in PBMCs from COVID-19 patients. Furthermore, as shown in Fig. 6b, the blood IL-6 levels in patients with SARS-CoV-2 are higher than healthy patients. Considering that the heterodimer of p65 iso5 with either p50 or p65 amplify the transcriptional activity of IL-6 and TNF-α promoters and the p65 iso5 mRNA levels are higher in COVID-19 subjects, p65 iso5 may be involved in the onset of cytokine storm and development of severe symptoms in patients with COVID-19. These results confirm the involvement of NF-kB in disease progression^41^.

### Identification of p65 iso5 protein in human liver tissue samples

**Figure 7:**
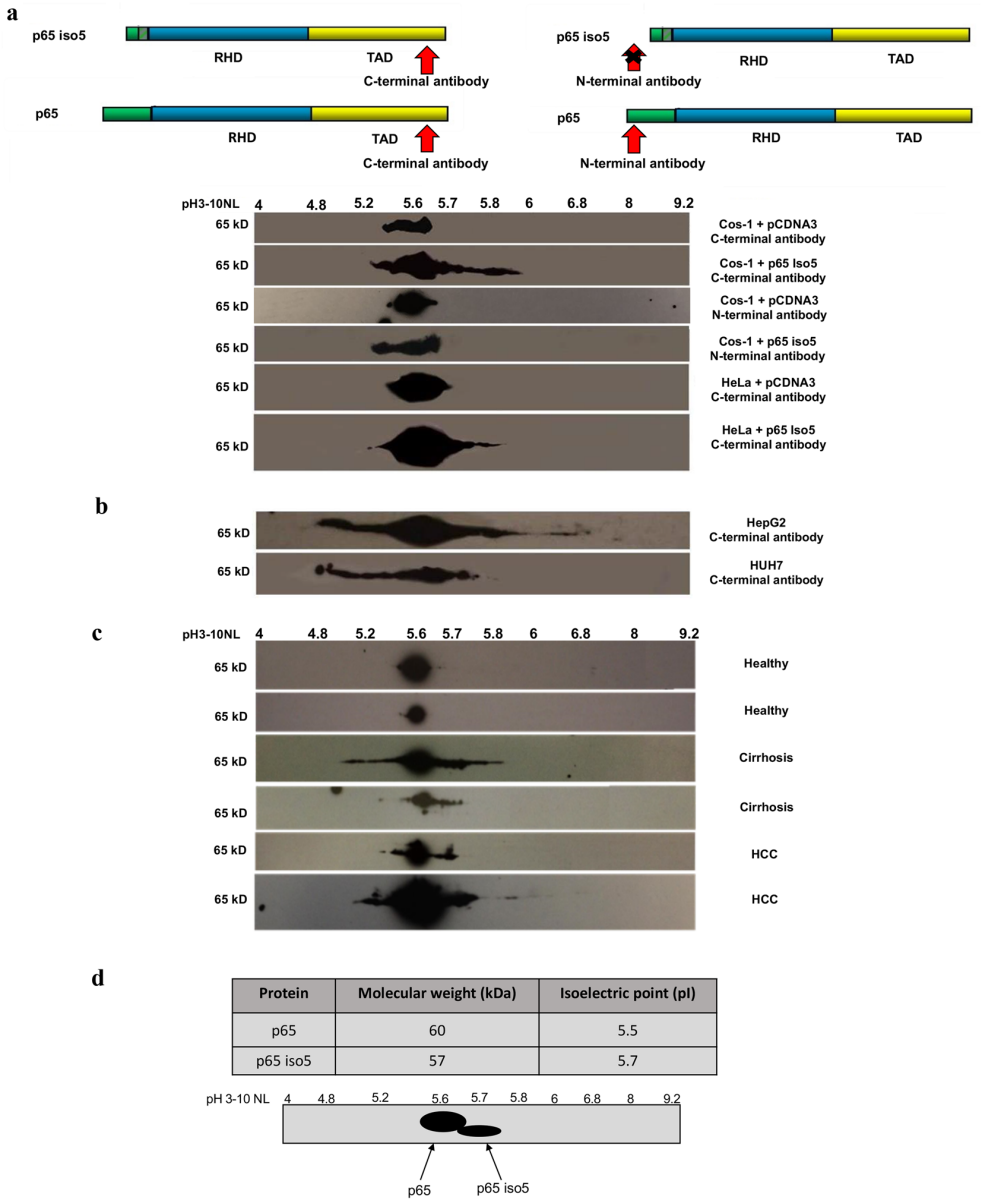
p65 and p65 iso5 protein expression by 2-DE in human liver samples, transfected cell lines and human liver carcinoma cells. (**a**) Expression of p65 and p65 iso5 proteins by 2-DE in Cos-1 and HeLa cells transfected with p65 and p65 iso5 proteins with specific antibodies for C-terminal and N-terminal epitope region. **(b**) Expression of p65 and p65 iso5 proteins by 2-DE in HepG2 and HUH7 hepatocarcinoma cell lines. **(c**) Electrophoretic pattern of the p65 and p65 iso5 proteins on human liver of healthy, cirrhosis, and hepatocellular carcinoma (HCC) samples. The p65 antibody is specific for an epitope at C-terminal region that is present both in p65 and in p65 iso5 proteins. **(d**) Theoretical Molecular weight (Mw) and Isoelectric point (pI) of p65 and p65 iso5. The values were calculated using Compute pI/Mw tool (ExPASy) and do not take into consideration the possible post-translational modifications (PTMs). Schematic representation based on the hypothetical pI and Mw of the p65 and p65 iso5 protein pattern on 2D-PAGE. The full length images of 2-DE are available in the “Supplementary Information” file.

In tumor cells, several molecular alterations could compromise the activation mechanism of NF-kB leading to genes deregulation involved in the control of cell cycle, apoptosis, cell migration or adhesion. Since alteration in some of these processes can determine cancer progression, it is clear that there is a connection between NF-kB and carcinogenesis^42,43^. Because NF-kB is involved in the regulation of inflammatory response, we analyzed the p65 protein profile in inflammation liver disease by coupling two-dimensional polyacrilacrylamide gel electrophoresis (2D-PAGE)^44,45^ and western blotting. As control, the expression profile of p65 iso5 protein was analyzed in transfected Cos-1 and HeLa cell lines with two different antibodies specific for the NH_2_ and COOH epitopes (Fig. 7a). Subsequently, we investigated the expression of p65 iso5 in the hepatocarcinoma cell lines HepG2 and HUH7 (Fig. 7b) and in liver extracts isolated from cirrhosis and HCC patients using healthy condition as control for the identification of p65 and p65 iso5 products (Fig. 7c). A more complex isoelectrofocusing pattern for p65 isoforms was shown in HCC and cirrhosis patients in comparison with control condition. The 2D-PAGE analysis, based on predicted isoelectric point (pI) and molecular weight (Mw), shows that these patterns (Fig. 7d) are compatible with the presence of the p65 iso5 and p65 protein in pathological samples, while only p65 is detected in healthy control samples (Fig. 7c). The functional characteristics of the protein encoded by this spliced mRNA are very different from the ones of the canonical p65. Such as the p65 wt, p65 iso5 mRNA is expressed in every mouse and human tissue tested but whilst the p65 wt protein is constitutive expressed in the liver^46^, p65 iso5 gene product is detected only under specific conditions associated with inflammation-related liver diseases. This is an additional different feature of p65 iso5 compared to p65 wt, that adds a further level of complexity to the regulation of NF-kB pathway.

## Discussion

Under physiological conditions, activation and resolution of inflammatory responses are regulated by a cascade of cellular events promoting the repair and healing. It is very well known that chronic inflammation can cause tissue damage and pathology in the organism as the development and progression of human cancers^47^. It is known that there is a cross talk between NF-kB and GR pathways and this is involved in pro- and anti-inflammatory response. NF-kB induces the transcriptional activation of genes involved in the stimulation of immune and inflammatory response. The GR, on the contrary, represses NF-kB and induces the expression of anti-inflammatory genes. Here we showed that exist a new splice variant of p65, named p65 iso5, that is transcribed starting by a new identified exon located upstream of the first known exon. This is the only p65 variant present both in human and mouse showing the importance of this gene product throughout evolution. This new isoform, in the presence of synthetic GC, enhances the GR-mediated anti-inflammatory response. Moreover, this protein, according to the partner with which it forms the dimer, is able to activate the promoter of some target genes in a different way compared to wild type. As demonstrated by docking simulation and BRET assay results, p65 iso5 is able to bind dexamethasone, showing a very different capacity in regulating the effects mediated by GCs. Our results strongly suggest that this isoform can contribute in a fine and more complex way to the inflammatory response. Since the anti-inflammatory response of GR is also mediated by transcriptional activation of its target genes^48^, the p65 iso5 interaction with dexamethasone could amplify and improve the GR-mediated anti-inflammatory response. Our results are consistent with the idea that the p65 iso5 expression is present in every tissue and cell line analyzed. The expression and the physical interaction between p65 iso5 and GR are critical for target genes transcriptional control. We hypothesize that this complex could control the GR’s anti-inflammatory actions in a locus specific manner. The study of the physical interaction between p65 iso5 and GR could highlight the importance of glucocorticoids to control the inflammatory response both in physiologic or pathological conditions. The discovery of a new p65 isoform with different biochemical property, compared with the wild type, and its different ability to activate a broad range of target genes can shed some light on the fine-tuning of inflammation resolution. In fact, we believe that the distinct features of p65 and p65 iso5 could provide a new ground to explain the complex and sometime opposite pro- and anti-inflammatory roles functions attributed to NF-kB^49^. In some studies, it has been observed that both the nucleocapsid protein and the spike protein of SARS-CoV were shown to induce pro-inflammatory cytokines via activation of the NF-kB pathway^50,51^ and that this has a key role in the SARS-CoV-2 infection^52,53^. Here we demonstrate that, p65 iso5 is overexpressed in the PBMC of COVID-19 patients and, in our knowledge, this is the first example showing a new gene product associated with the pathological profile of Sars-CoV-2 infection. Considering the ability of the new isoform p65 iso5 to bind dexamethasone and to regulate the glucocorticoid responses in the opposite way of the wild type, p65 iso5 could play a therapeutic role in alleviating the severe form of COVID-19. In fact, the role of steroids in these patients is to inhibit the expression of certain molecules involved in pneumonia associated inflammatory response^54^. We propose a model in which p65 iso5, according to the homo and heterodimeric complex, can be considered as a stronger pro-inflammatory response. Indeed, this new isoform can dimerize with either p65 or p50 to coordinate an onset phase of different mediators. In addition, p65 iso5 could interact with GR through the formation of a complex and, after treatment with glucocorticoids, amplify the transcription of its target genes involved in the anti-inflammatory response (Supplementary Fig. S3). We also show that p65 iso5 protein expression is associated with inflammation-related liver diseases. In fact, the p65 iso5 protein is present only in the liver samples of patients suffering from cirrhosis and HCC. This result strongly suggests a role for p65 iso5 to contributing to the onset of these inflammatory liver diseases identifying this new protein as potential new therapeutic target for specific GC ligands that could improve clinical and long-term treatments with GCs.

## Methods

### Animal and human samples

All animal procedures were conducted following the ARRIVE (Animals in Research: Reporting in Vivo Experiments) guidelines and standard ethical guidelines (European Communities Directive 2010/63/EU). The experimental protocols were approved by the animal welfare committee of the University of Palermo and authorized by the Ministry of Health (Rome, Italy). 8 weeks-old male C57BL/6JCO Mus musculus were housed in individual cages in a controlled environment (temperature, humidity light/dark cycle). Animals 1–3 h after the beginning of the light phase, were sacrificed following anesthesia with CO_2_. After sacrifice, brain structures and/or organs were dissected and rapidly frozen in liquid nitrogen and then stored at − 80 °C until protein or RNA extraction. For blood donor samples, the study was conducted after obtaining COVID Unit approval (Department of Internal Medicine, Hospital of National Relevance and High Specialization ARNAS Civico, Di Cristina-Benfratelli) and the patient informed consents for research studies. The study on human liver samples was conducted after obtaining the IRCCS-ISMETT approval (Istituto Mediterraneo per i Trapianti e Terapie ad alta specializzazione—Department of Laboratory Medicine and Advanced Biotechnologies) and the patient informed consents for research studies. We confirm that the human samples were used in accordance with national guidelines and regulations.

### Cell cultures and transfections

COS-1 cells (ATCC CRL-1650TM) were grown on DMEM (high glucose) without antibiotics supplemented with 10% fetal bovine serum at 37 °C under 5% CO_2_. HeLa cells (ATCC CCL-2TM) were grown at 37 °C under 5% CO_2_ on RPMI without antibiotics supplemented with 5% heat inactivated fetal bovine serum. All the transfection experiments were performed using Polyfect Reagent (Qiagen) following manufacturer’s instructions. For western blot experiments, cells were plated and transfected 24 h later with the appropriated plasmid. For luciferase experiments 3 × 10^5^ HeLa cells/well were plated on six wells dishes and transfected 24 h later with the appropriate concentrations of the specified plasmid. All the experiments were performed in triplicate and replicated twice. The quantity of plasmid for each well was kept constant by adding appropriate amounts of the corresponding empty plasmid.

### Isolation of peripheral blood mononuclear cells

Peripheral Blood Mononuclear Cells (PBMC) have been isolated from venous blood samples of 21 patients affected by SARS-CoV-2 and 21 healthy subjects, for a total of 42. PBMC were isolated from whole blood through density-gradient centrifugation, by using Ficoll Paque (GE Healthcare), following manufacturer’s instructions.

### RNA extraction and RT-PCR

Mouse or human total RNA was extracted using the RNA NOW reagent (Ozyme) or TRIzol™ Reagent (Invitrogen™). All the experiments were performed in triplicate. The RNA (1 or 2 μg) was reverse transcribed with the using Oligo (dT) primers, RNAsin RNase Inhibitor, M-MLV Reverse Transcriptase and dNTP from Promega. After extraction the RNA was quantified with a spectrophotometer and electrophoresed on agarose/formaldehyde gel to control the quality of the samples. A dilution (1:20) of the RT reaction was used for PCR reaction with specific primers for exon -1/exon 2, exon − 1/exon 0, exon 0/exon 2 (forward exon − 1 primer 5′-GGCCTGGGCCTCTCCCTGCGCAGGGCGAATG-3′; forward exon 0 primer 5′-CTTTAGCGCGCCGTGGGCTCAGCTGCGA-3′; reverse exon 2 primer 5′-ATGGTGGGGTGTGTCTTGGTGGTATCTG-3′; reverse exon 0 5′-TCCCGGGGGCGGGGCCGGGGTCGCAGCT-3′). One–fifth of the reaction was analyzed on agarose (2%) gels. Nested PCR (a 1:200 dilution of the first PCR reaction) was used to specifically amplify the entire p65 iso5 mRNA product from heart, brain, spleen, lungs, liver, kidneys, skeletal muscle, testicles, thymus of mouse and brain, liver, peripheral blood mononuclear cell (PBMC) of human RNA. In the first reaction, for the mouse sample, specific primers for exon − 1/3′UTR (untranslated region) were used (forward exon − 1 primer 5′-GGCCTGGGCCTCTCCCTGCGCAGGGCGAATG-3′; reverse 3′UTR primer 5′-AGGCTTCAGTGCCCTGAAACCTGGTG-3′). One tenth of the first PCR product was diluted in 250 μl of distilled water and 5 μl were used for the second reaction of the nested PCR exon − 1/exon 0, exon − 1/exon 2, exon − 1/exon 5, exon − 1/exon 7, exon − 1/exon 9, exon − 1/exon 10 (forward exon − 1 primer 5′-ATGGCCGCAGCCTGCCACCAGCCCTGGC-3′; reverse exon 0 primer 5′-TCGCAGCTGAGCCCACGGCGCGCTAAAG-3′; reverse exon 2 primer 5′-ATGGTGGGGTGTGTCTTGGTGGTATCTG-3′ reverse exon 5 primer 5′-GGTTATCAAAAATCGGATGTGAG-3′; reverse exon 7 primer 5′-GGAGCCTCGTGCCTCCCAGCCTGG-3′; reverse exon 9 primer 5′-GGCTTGGGGACAGAAGTTGAGTTT-3′; reverse exon 10 5′-CTCTGAGCAGGGTCGCTGTCAGCACC-3′). Human brain total RNA (Origene) or liver or PBMC total RNA (1 or 2 μg) were isolated and reverse transcribed; a dilution (1:20) was used in the first PCR reaction with specific primers for exon − 1/3′UTR (forward exon − 1 primer 5′-GGAGGGCCTCAGTCGTCCCATC-3′; reverse 3′UTR 5′-AGAATCCGTAAGTGCTTTTGGAGG-3′). The second reaction of the nested PCR (a 1:200 dilution of the first PCR reaction) with specific primers for exon − 1/exon 5, exon − 1/exon 7, exon − 1/exon 9, exon − 1/exon 10 (forward exon − 1 primer 5′-TCTTGATGCACTGTCAGGCTG-3′; reverse exon 5 primer 5′-GGACAGGCGGCAGGCGGAGGGGCC-3′; reverse exon 7 primer 5′-ATGGGCTCACTGAGCTCCCGGTC-3′; reverse exon 9 primer 5′-AGCTGCGGGAAGGCACAGCAATGC-3′; reverse exon 10 5′-AGCTGATCTGACTCAGCAGGGC-3′). Nested PCR product from human and mouse samples were loaded on agarose (1%) gels. PCR products were cloned and the specificity was confirmed by sequencing. At least three different clones for each PCR product were sequenced.

### Real-time quantitative RT-PCR (qPCR)

The oligonucleotides used for qPCR were designed based on the gene sequences achieved from the Nucleotide database NCBI (GeneBank) and validated for absence of secondary structures, self-dimers as well as primer efficiency and specificity. The p65 sequences 5′AATGGCTCGTCTGTAGTGCACGC 3′ and 5′CCGGGAAGATGAGGGGGAAC 3′ and for p65 iso5 5′ GAAATCCCCTAAAAACAAA 3′ and 5′ CCGGGAAGATGAGGGGGAAC 3′. For each RT-qPCR reaction were used 10 μl of ExcelTaq™ 2X Fast Q-PCR Master Mix (SYBR, ROX) (SMOBIO), 10 pmol/μl of the respective forward and reverse primer, 50 ng of cDNA and RNase-free H_2_O (GIBCO) to a final volume of 20 μl. All cDNA samples were tested as three replicates for housekeeping gene on the same 96 well PCR plate replicate in 40 cycles (95 °C for 20 s, per cycle 95 °C for 3 s, 60 °C for 30 s) to reduce possible variations on relative housekeeping gene. Non-template controls and reverse transcription controls were additionally performed. For qPCR a StepOnePlus™ Real-Time PCR System (Applied Biosystems, Thermo Fisher) was used in with 96-well PCR plates covered with MicroAmp™ Optical Adhesive Film (Applied Biosystems, Thermo Fisher).

### IL-6 measurements

The serum and plasma IL-6 levels were assayed through the chemiluminescence (EIA) method on Cobas 8000 analyzer (Roche Diagnostics GmbH).

### In vitro transcription/translation and electrophoretic mobility shift assay (EMSA) of p65 and p65 iso5

The mouse p65 iso5 with 169 bp of exon − 1 and the p65 cDNA were cloned into pBluescript vector (Stratagene). Uncut plasmid DNAs were transcribed and translated using TNT kit (Promega) following manufacturer’s instructions. The in vitro unlabelled translated proteins were incubated with a double-stranded ^32^P-labelled oligonucleotide probe containing the specific recognition sequence for NF-kB (5′-AGTTGAGGGGACTTTCCCAGGC-3′), and analyzed on non-denaturing 5% acrylamide gel. Additionally, to the labelled NF-kB probe an unlabelled oligonucleotide was added in excess (100:1) when appropriate.

### Luciferase assay

24 h after plating in appropriate growth medium, HeLa cells were transiently co-transfected with 500 ng of reporter plasmids pNFkB-luc or pGRE-luc (Clontech) or pIL6-651 bp-luc or TNFα-luc and with 25 ng of the phRL-TK (Promega) vector encoding the Renilla luciferase as internal control. For the experiment with the pNFkB-luc reporter, cells were cotransfected with plasmid encoding either the human p65 or p65 iso5 cDNA (250 ng) under the SV40 promoter. The enzymatic activity was measured 24 h after transfection. For the experiment with the pGRE-Luc reporter, cells were cotransfected with either p65 or p65 iso5 plasmids (500 ng). The day after transfection, the cells were treated with 5 × 10^–8^ M of the GR agonist Dexamethasone (Sigma) in supplemented fetal bovine serum medium treated with Charcoal–dextran (Hyclone) and the enzymatic activity was measured 24 h later. For both the pNFkB-luc and pGRE-luc experiments the total amount of DNA (1.5 μg) for each transfection was kept constant by adding the appropriate quantity of empty plasmid. The enzymatic activity of the two individual reporter enzymes (Firefly and Renilla) was measured in 20 μl of cell lysate with the Dual-Luciferase Reporter Assay System (Promega) according to manufacturer’s instruction using a luminometer LUMAT LB 9507 (Berthold Technologies). All the experiments were performed in triplicates and replicated twice.

### In silico interaction and docking studies of p65 iso5 and dexamethasone

The amino acid sequence of our p65 iso5 was used to perform a BLAST research in order to recover a PDB structure of a template. The amino acid sequence of the human p65 iso5 was obtained starting from the amino acid 32 of sequence of p65 wt UniProtKB -Q04206 (https://www.uniprot.org/uniprot/Q04206). The 3D structure of p65 iso5 was built with Modeller v9.8^35^ using as template the p65 of the Protein Data Bank crystal complex IkappaBalpha/NF-kappaB (PDB: 1NFI; https://www.rcsb.org/). In order to select the lowest energy structure, 20 different models were computed with Modeller v9.8, and Discrete Optimized Protein Energy function was evaluated. Our model of the p65 iso5 differs from the p65 wt by the mere lack of the first 31 residues, possessing a homology of 100% with the template, making the model as good as the crystalized structure of the template (Resolution: 2.70 Å). Chimera v1.11.2^36^ was adopted to visualize the model and to compare it with the wild-type structure by structural superimposition and distance analysis. The dexamethasone–GR human structure (PDB: 4UDC) was also structurally aligned to the p65 mutant with the function MatchMaker in order to compare the residues involved in the binding of the dexamethasone. The Rigid Molecular Docking of dexamethasone to the wt p65 and to the modelled p65 mutant was performed with Autodock v4.2^37^. The dexamethasone coordinates were obtained from the PDB structure in complex with the GR. The docking analysis on the p65 iso5 and on the p65 wt was performed over a 3D grid box (dimensions 60 × 60 × 60 unit in number of grid points to occupy more potential areas of binding; grid spacing 0.375 Å) created using Autogrid v4.2. A total of 1500 runs of Lamarckian genetic algorithm were performed, with an initial population of 300 conformations, a cut off of 27,000 generations, and with rates of mutation and crossover set to 0.02 and 0.8, respectively. The final solution was characterized by the lowest binding energy.

### BRET (Bioluminescence Resonance Energy Transfer) ligand binding assay

Cos-1 cells were seeded at a density 200,000 cells on 6-wells tissue culture plates containing DMEM (high glucose) (GIBCO) and 10% fetal bovine serum heat inactivated (GIBCO) incubate at 37 °C, 5% CO_2_. The next day the cells were transfected with a BRET constructs (human GR, p65 and p65 iso5 cDNA fused in frame C-terminal with Rluc) using PolyFect Trasfection Reagent (Qiagen) according to the manufacturer’s instructions. 24 h post-transfection cells were harvested using trypsin; 50,000 cells were plated on 96-well plates with white bottom, incubated for 30’ and subsequently treated with Dexamethasone Fluorescein (Dex-FITC) [5 μM] (Thermo Fisher) for 4 h. Later a final concentration of 5 μM Coelenterazine h (Sigma) was added. The 530 nm and the 495 nm emission fluorescence was immediately read at room temperature using GloMax Discover Microplate Reader (Promega). The raw BRET ratio was calculated by dividing the 530 nm emission by the 495 nm emission. All the experiments were performed in triplicates and replicated twice.

### Sample preparation 2-DE and western blot analysis

The human liver samples have been delivered frozen in cryotubes by 2 ml and stored − 80 °C. All procedures were performed at 4 °C on ice. Approximately 25 mg of liver tissue were collected in 500 μl lysis buffer (7 M Urea, 2 M Thiourea, 30 mM CHAPS, 2% Triton X100, 39 mM TRIS pH 8.8, 65 mM DTT, 1 mM Na_3_VO_4_, 1% protease inhibitor cocktail). The tissues have been homogenized 20 times with Dounce (Pestle A), transferred in 2 ml microtubes and centrifuged at 10,000×*g* for 10′ a 15 °C. The supernatant containing the total proteins has been recovered. Protein concentration was determinate by Bradford assay (Bio-Rad). For the first dimension, IEF (isoelectric focusing), 1.5 mg of proteins were loaded in to Immobiline Dry Strips Gel, 18 cm, pH 3–10 nonlinear (GE Healthcare) according to the manufacturer's protocol. The samples were mixed with rehydration buffer (8 M Urea, 4% CHAPS, 1% DTT, 1% bromophenol blu) containing DTT (20 mM) in a final volume of 340 μl, and IPG buffer pH 3–10 NL (0.6%) (GE Healthcare) was added. Strips were rehydrated for 1 h at 20 °C. IEF was performed at 20 °C on an Ettan IPGphor 3 IEF System (GE Healthcare) and low voltage (30 V) was applied for 10 h. Successively, the voltage was gradually increased to 8000 V over 6 h and was maintained at the level until a total of 76,000 Vh. Focused IPG strips were soaked in the equilibration buffer (6 M Urea, 50 mM TRIS pH 6.8, 2% SDS, 30% glycerol) with DTT (120 mM) for 10′ and subsequently with iodoacetamide (134 mM). After equilibration, proteins were separated in the second dimension using ETTAN DALTAsix Large Vertical System (GE Healthcare). The strips were placed on top of the 8% gel of the second dimension (gel size 160 × 200 × 1 mm) locked with agarose 0.5% with bromophenol blue (0.5%) prepared in Electrophoresis buffer (0.24 M TRIS, 1.9 M glycine) and run under denaturing at a constant power of 40 mA/gel. A cooling system provided constant 20 °C running temperature. Finished the run, the proteins were transferred to nitrocellulose membrane (Amersham Protran 0.45 NC nitrocellulose Western blotting membranes GE Healthcare) overnight at 4 °C, in Tris/glycine/methanol 20% buffer at 50 V. The membranes were then blocked for 30′ in a milk solution (5% dry powdered non-fat milk, PBS 1×, Tween 20 0,1%, with 100 μl/l of protease inhibitor cocktail) at room temperature and incubated for 2 h in a milk solution, with NF-kB p65 (G-8) (sc-398442) or NF-kB p65 (c-20) (sc-372) antibody dilution 1:5000 or NF-kB p65 (A) antibody (sc-109) (Santa Cruz Biotechnology) dilution 1:5000. Then the membranes were washed 2 times for 7′ with PBS 1×, Tween 20 0,1% and incubated for 1 h at room temperature in a milk solution containing anti-rabbit IgG-HRP secondary antibody (sc-2004 Santa Cruz) dilution 1:4000 when used NF-kB p65 (A) or NF-kB p65 (c-20) antibody and with anti-mouse m-IgGκ BP-HRP secondary antibody (sc-516102 Santa Cruz) dilution 1:4000 when used NF-kB p65 (G-8) antibody. The membranes were again washed three times for 5′ with PBS 1×, Tween 20 0.1% and once in PBS 1× alone. The signal was detected using ECL Prime (GE Healthcare) following the manufacturer's protocol.

### Statistical analysis

All data (mean ± sem) were analyzed by a Student’s t-test (two tailed) or by one-way ANOVA followed by Newman Keuls post hoc comparisons when appropriate.

### Immunofluorescence

Cos-1 cells were transfected with p65 and p65 iso5 proteins on eight-well chamber slide (Biosigma) with DMEM (high glucose) (GIBCO) supplemented with 10% heat inactivated fetal bovine serum. 24 h later, cells were fixed with 4% paraformaldehyde in PBS for 10′ at room temperature and permeabilized with 0.1% saponin; 0.05% NaN_3_ for 30′ at room temperature and washed twice with incubation buffer (0.5% BSA; 0.1% saponin; 0.05% NaN_3_). Immunostaining was performed with mouse-anti NF-kB p65 (L8F6) monoclonal PE conjugate (Cell Signaling) (1:50 dilution) for 1 h 30′ at room temperature and washed twice with incubation buffer. Nuclei were counterstained with 1 μg/ml of the nuclear stain Hoechst 33342 (Invitrogen) for 10′ at room temperature. Images were taken with a digital Leica DMRA 2 microscope and 20× magnification.

### Fluorescence microscopy

Cos-1 cells were transfected with GFP-p65 and GFP-p65 iso5 proteins on eight-well chamber slide (Biosigma) with DMEM (high glucose) supplemented with 10% heat inactivated fetal bovine serum. 24 h later cells were fixed with 4% paraformaldehyde in PBS for 10′ at room temperature and washed twice with PBS 1×. Nuclei were counterstained with 1 μg/ml of the nuclear stain Hoechst 33342 (Invitrogen) for 10′ at room temperature, the images were taken with digital Leica DMRA 2 microscope and 40× magnification.

### Human liver and blood donor samples

Liver tissue and blood samples were collected from Caucasian patients. The liver samples were collected from a cohort of 14 patients (5 healthy subjects, 7 with cirrhosis and 2 with hepatocellular carcinoma), undergoing liver surgery at the Department of Laboratory Medicine and Advanced Biotechnologies, IRCCS-ISMETT (Istituto Mediterraneo per i Trapianti e Terapie ad alta specializzazione), Palermo, Italy. All tissue samples had been examined by a pathologist, and only healthy, cirrhotic, hepatocellular carcinoma histologically diagnosed liver tissues were used and liver proteins from every patient had been extracted. Blood samples from 42 patients (21 health, 21 SARS-CoV-2) were collected at the COVID Unit, Department of Internal Medicine, Hospital of National Relevance and High Specialization ARNAS Civico, Di Cristina, Benfratelli Palermo, Italy. Written informed consent was obtained from each patient studied.

## Supplementary Information

Supplementary Information.

Supplementary Video S1.

Supplementary Video S2.

## Data Availability

All data concerning the new exon sequences published on this study are deposited on data bank and will be available on NCBI gene bank after the possible acceptance of publication. p65 iso5 (GenBank accession number MN508965), p65 isoform 3 (p65 iso3) (GenBank accession number MN508964).
