# Supplementary material for: A new p65 isoform that bind the glucocorticoid hormone and is expressed in inflammation liver diseases and COVID-19
Source: Sci Rep. 2021 Nov 25;11:22913. doi: 10.1038/s41598-021-02119-z (PMC8617276; doi:10.1038/s41598-021-02119-z)
Supplement: Supplementary file 1 — Supplementary Information. [file 41598_2021_2119_MOESM1_ESM.docx]

Supplementary Fig. S1

**
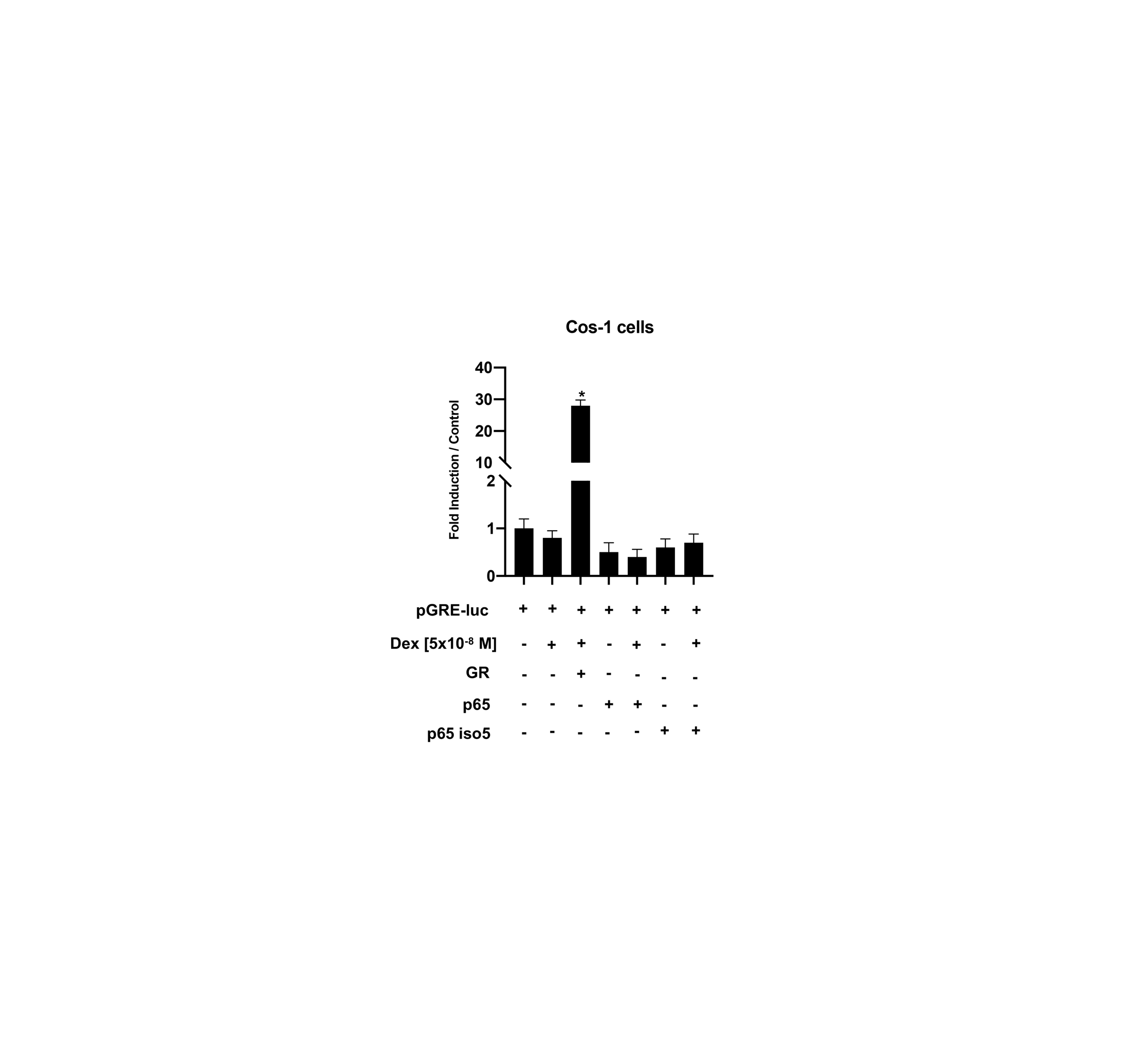
**

**Supplementary Fig. S1. p65 iso5 transcriptional activity on GRE-luc promoter in Cos-1 cells treated with GCs.** Activity of p65 iso5 protein in Cos-1 cell line treated with GCs. Cos-1 cells were cotransfected with a luciferase reporter driven by promoter GRE-luc and the indicated plasmids. Cells were maintained in DMEM supplemented with Charcoal-dextran (CD) Fetal Bovine Serum (FBS). Data information: In A and B, data are presented as mean ± SEM. *P<0.001, in comparison to cells transfected with reporter plasmid.

Supplementary Figure S2

**
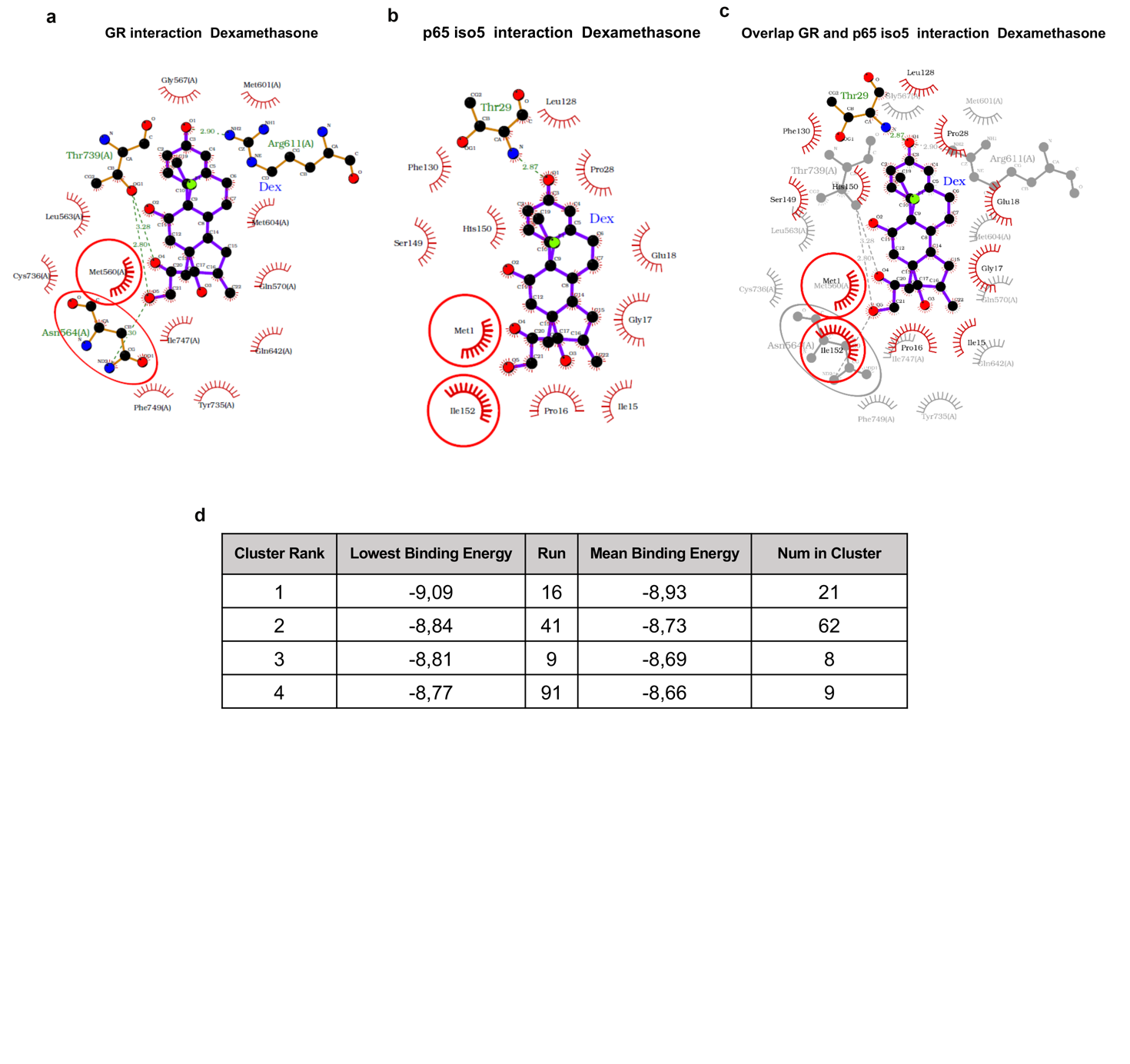
**

**Supplementary Fig. S2. Docking simulation of GR and p65 iso5 proteins with dexamethasone.** (A, B, C) Dexamethasone-GR, dexamethasone-p65 iso5 and Overlap of GR and p65 iso5 interaction with dexamethasone. The docking simulation are performed using LIGPLOT v 4.5.3; in red circles overlapped amino acids of the GR (Met560 and Asn564) and p65 iso5 (Met1 and Ile152) proteins. (D) Number of distinct conformational clusters found using an RMSD-tolerance of 2.0 A.

Supplementary Figure S3


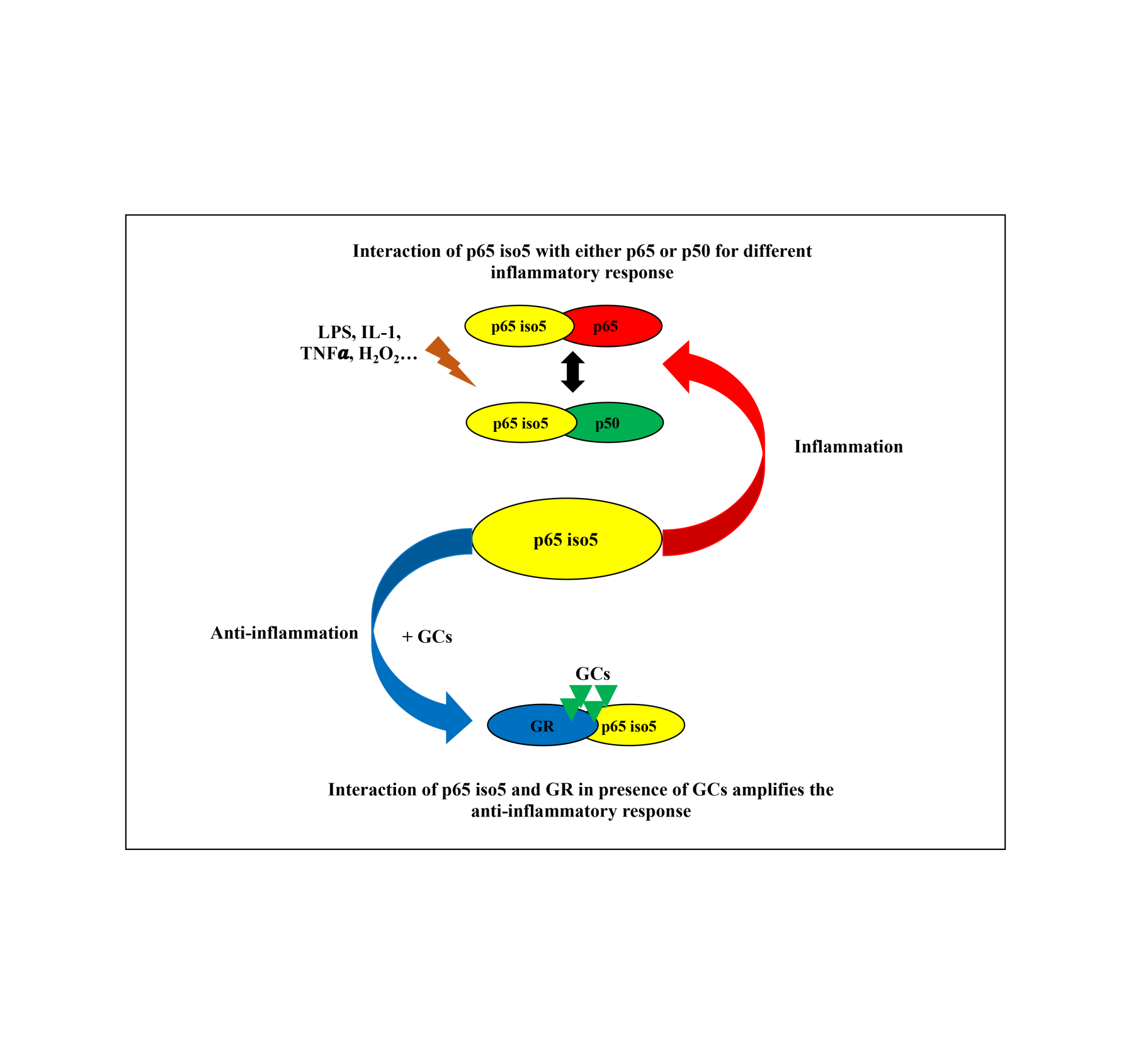


**Supplementary Fig. S3**. Proposed model for the involvement of the p65 iso5 protein in the regulation of the inflammatory response mediated by NF-κB complex.

Full length gel images Figure 2a


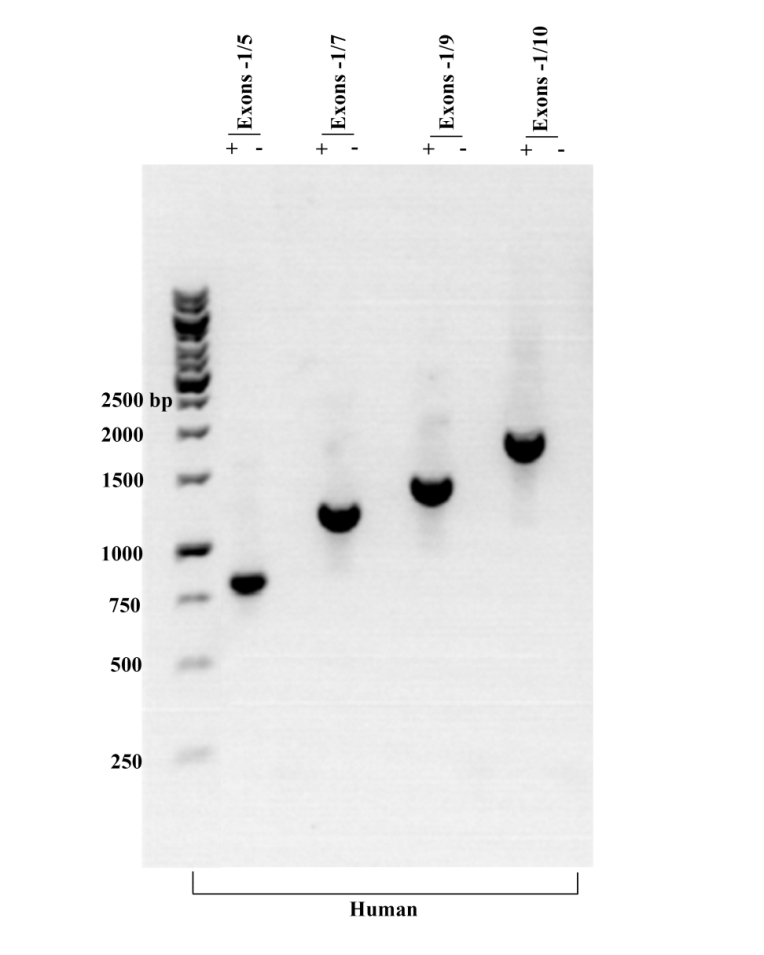


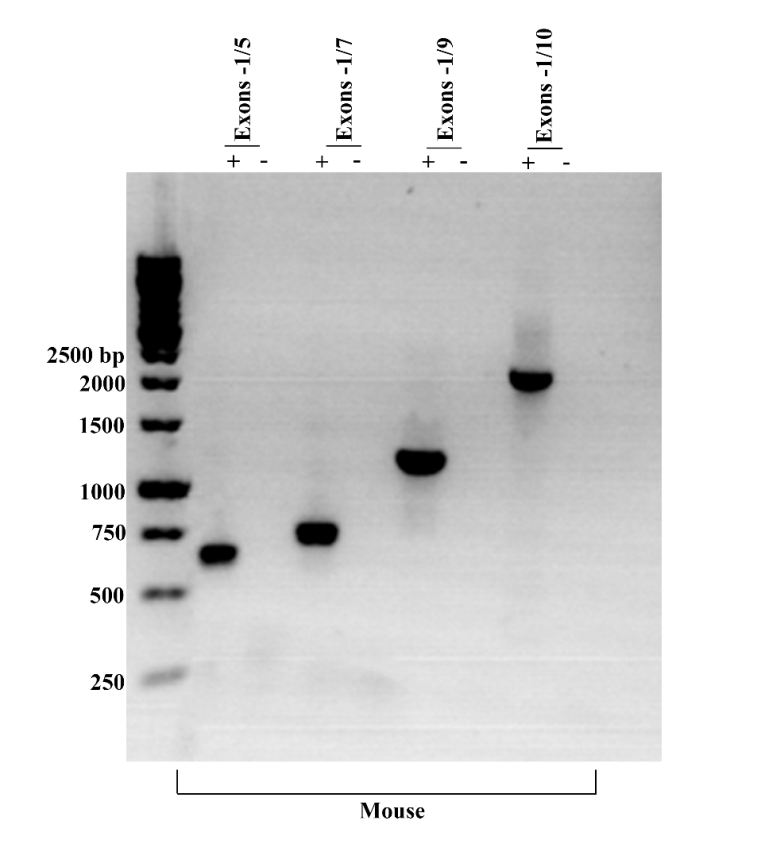


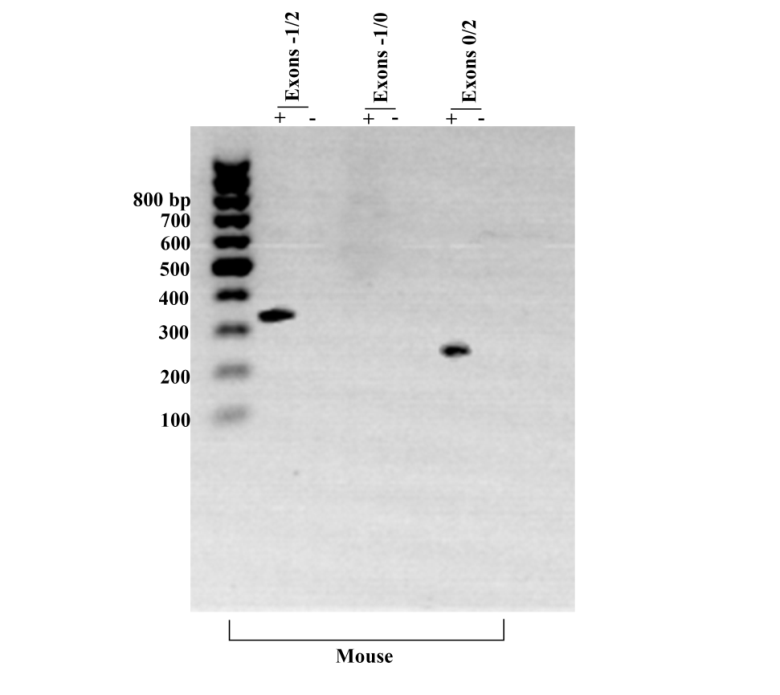


Full length gel image Figure 2b


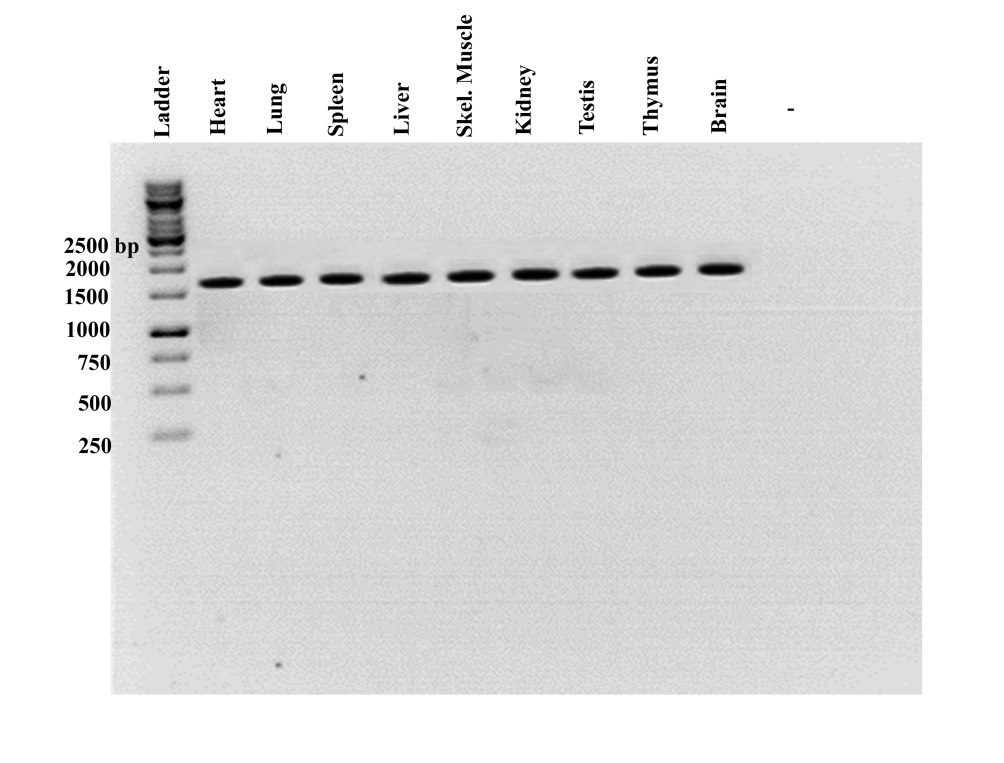


Full length gel image Figure 3a


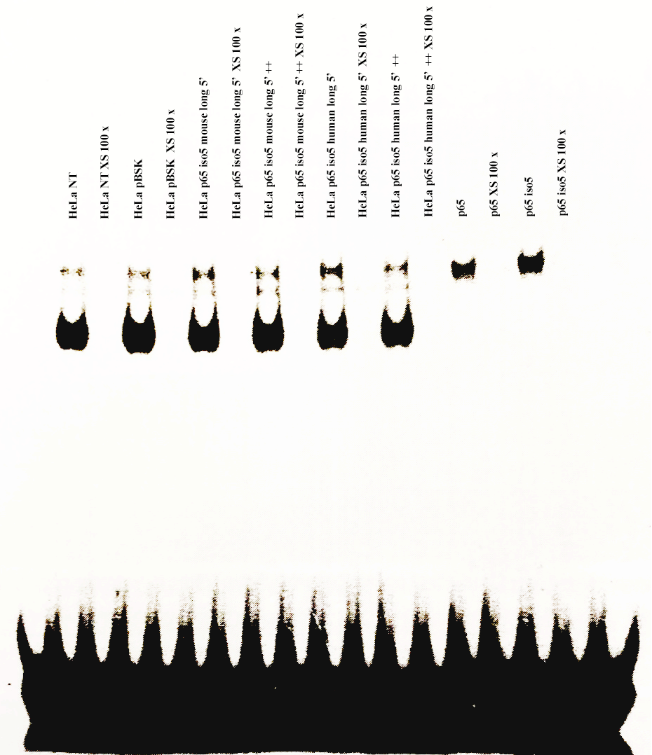


Full length gel image Figure 3b. The membrane has been cut prior exposition.


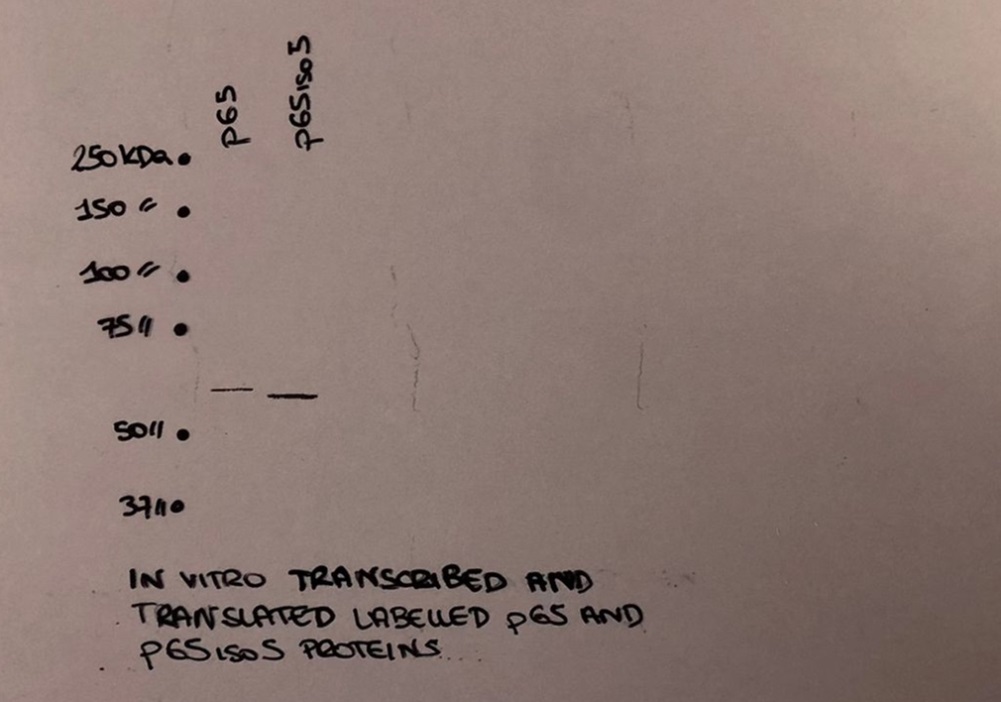


Full length gel image Figure 3b (longer exposure)


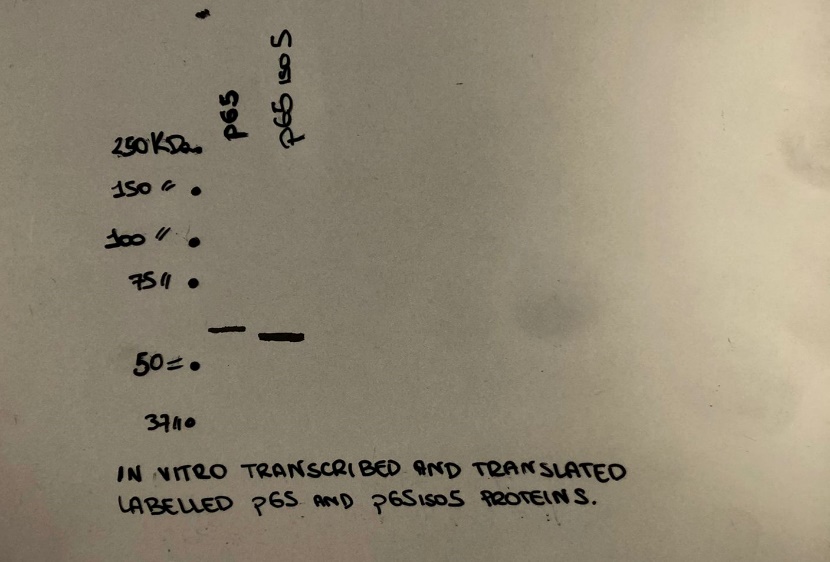


Figure 7. Full length gel images

**Cos-1 + pcDNA3 C-terminal**


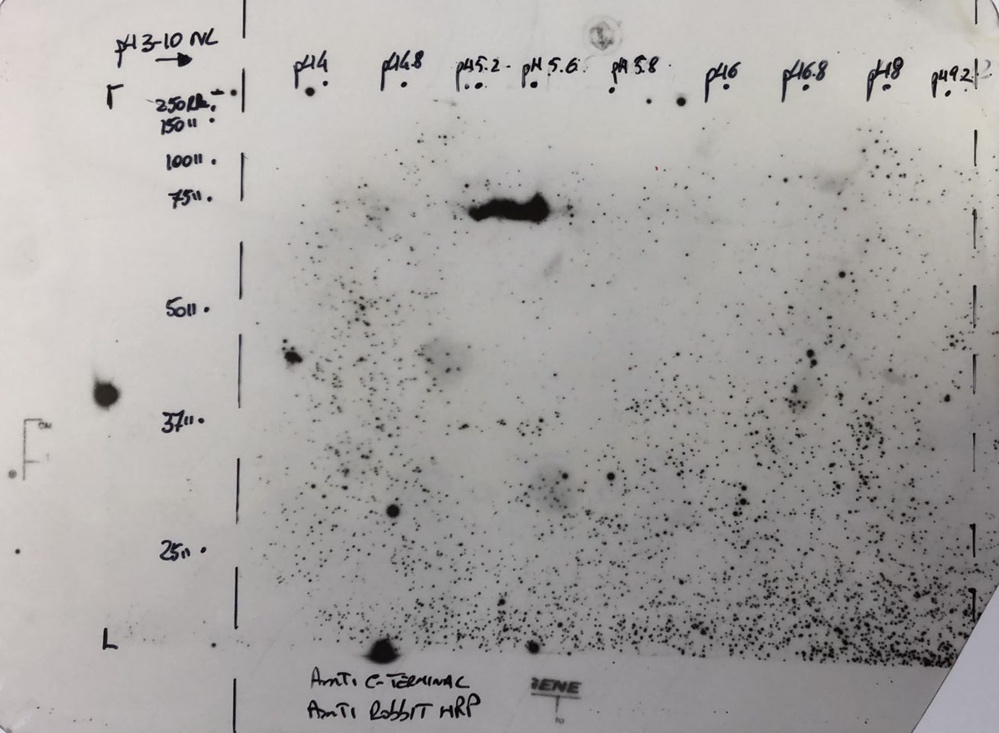


**Cos-1 + p65 iso5 C-terminal**

**
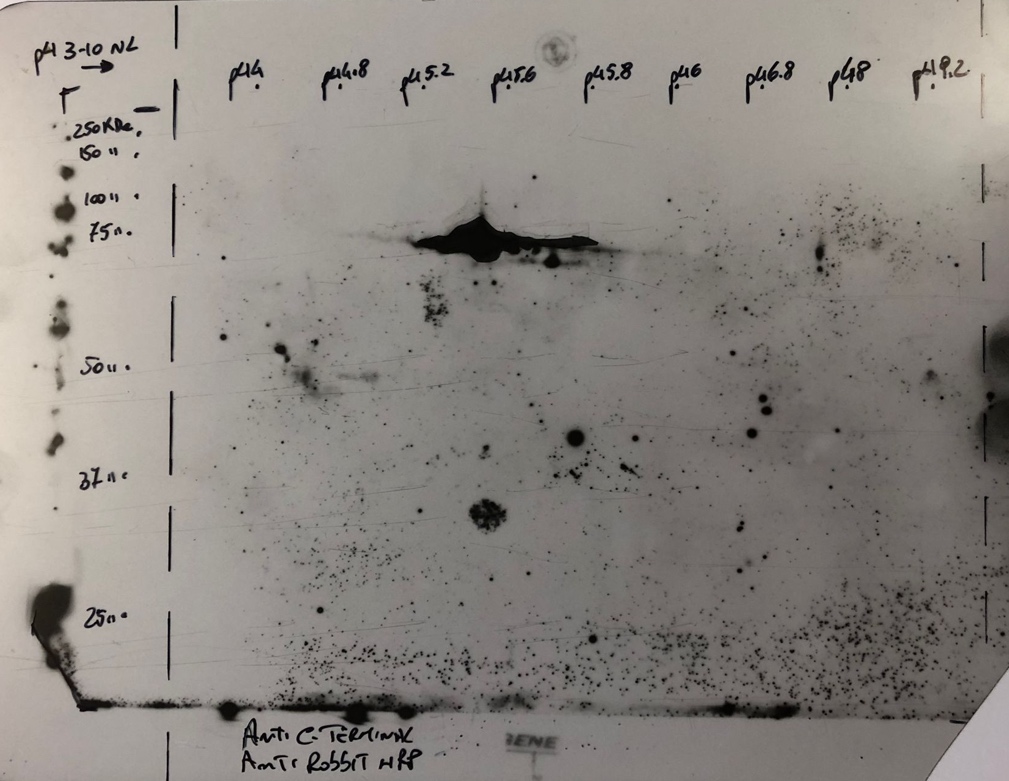
**

**Cos-1 + pcDNA3 N-terminal**

**
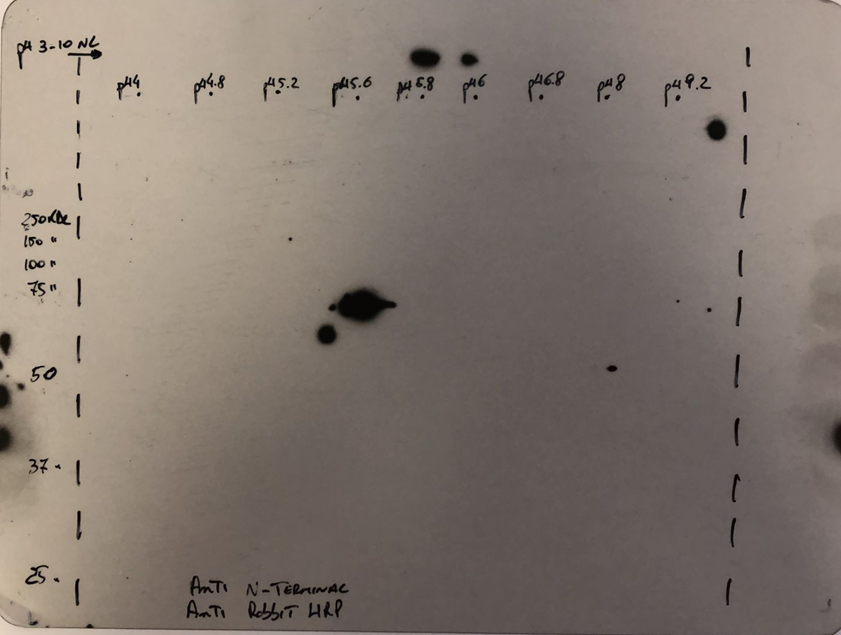
**

**Cos-1 + p65 iso5 N-terminal**

**
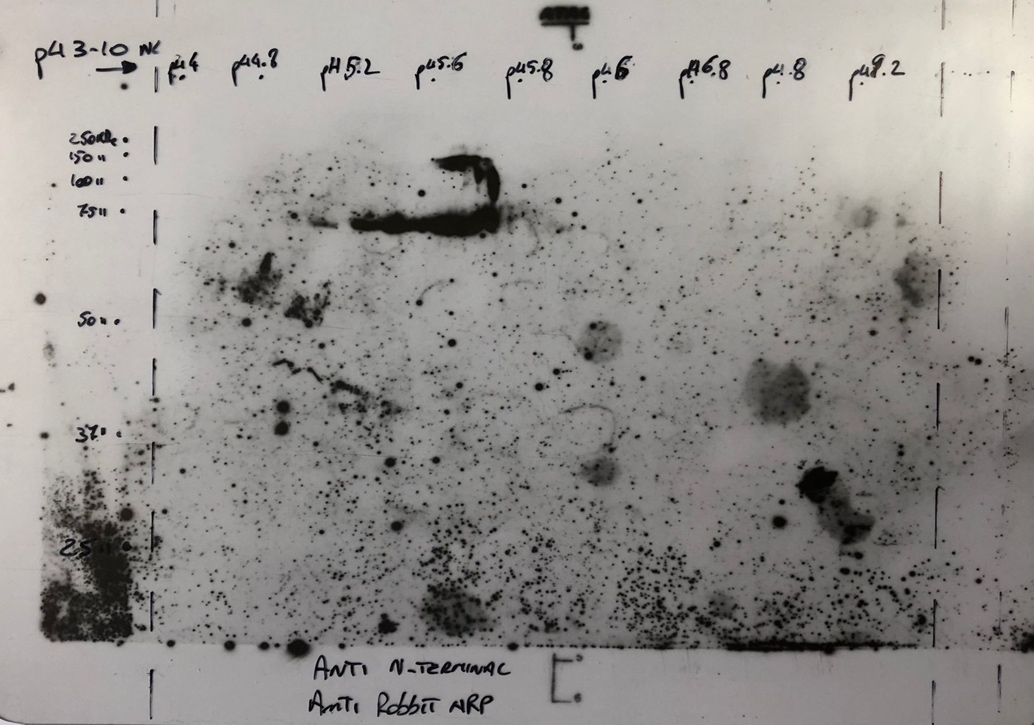
**

**HeLa + pcDNA3 C-terminal**

**
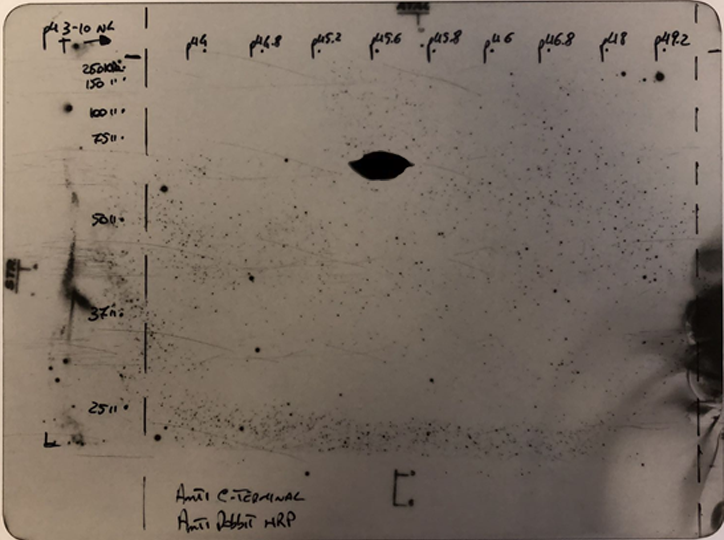
**

**HeLa + p65 iso5 C-terminal**

**
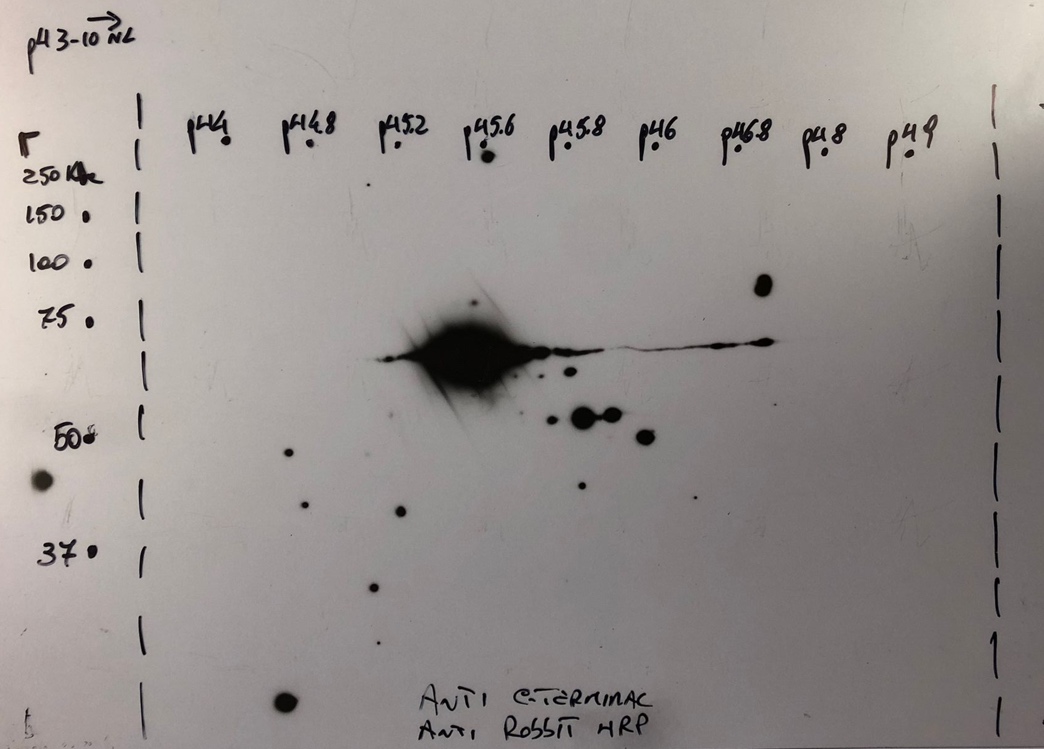
**

**HepG2**


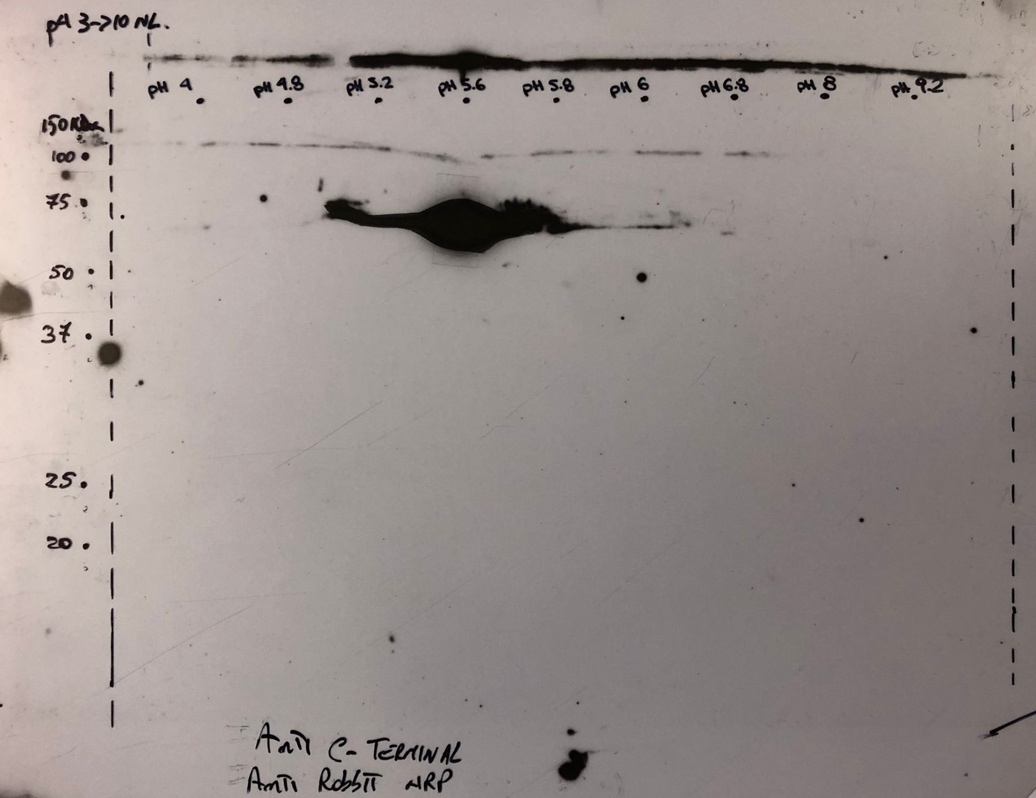


**HUH7**

**
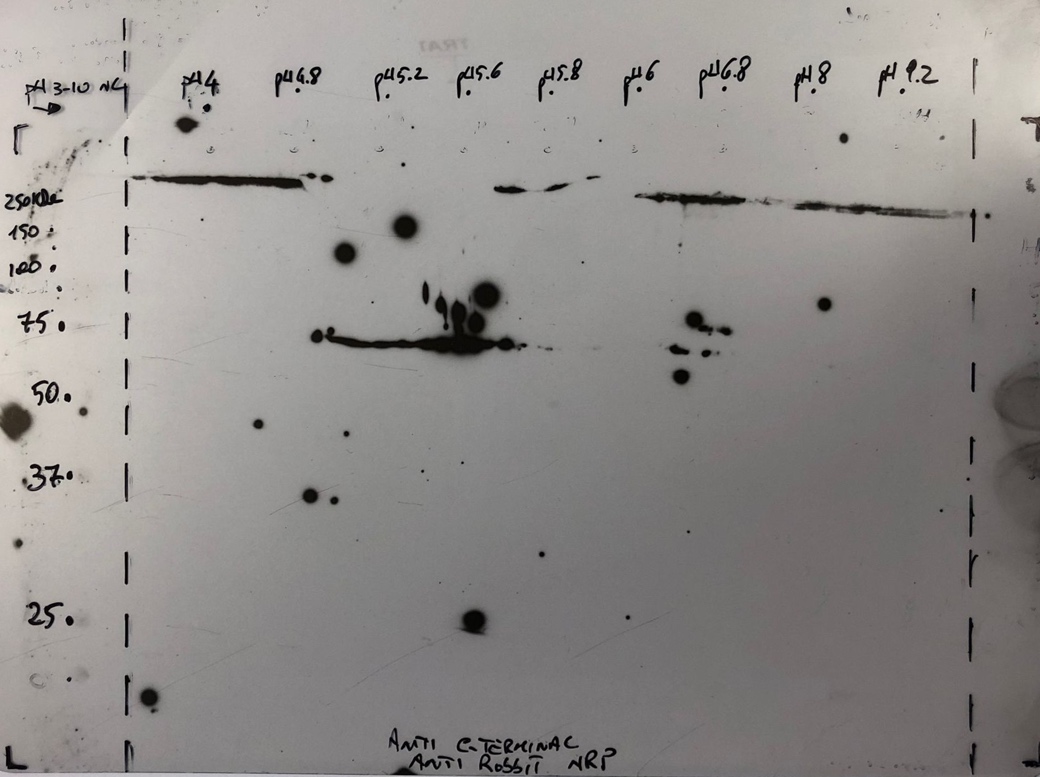
**

**Healthy**

**
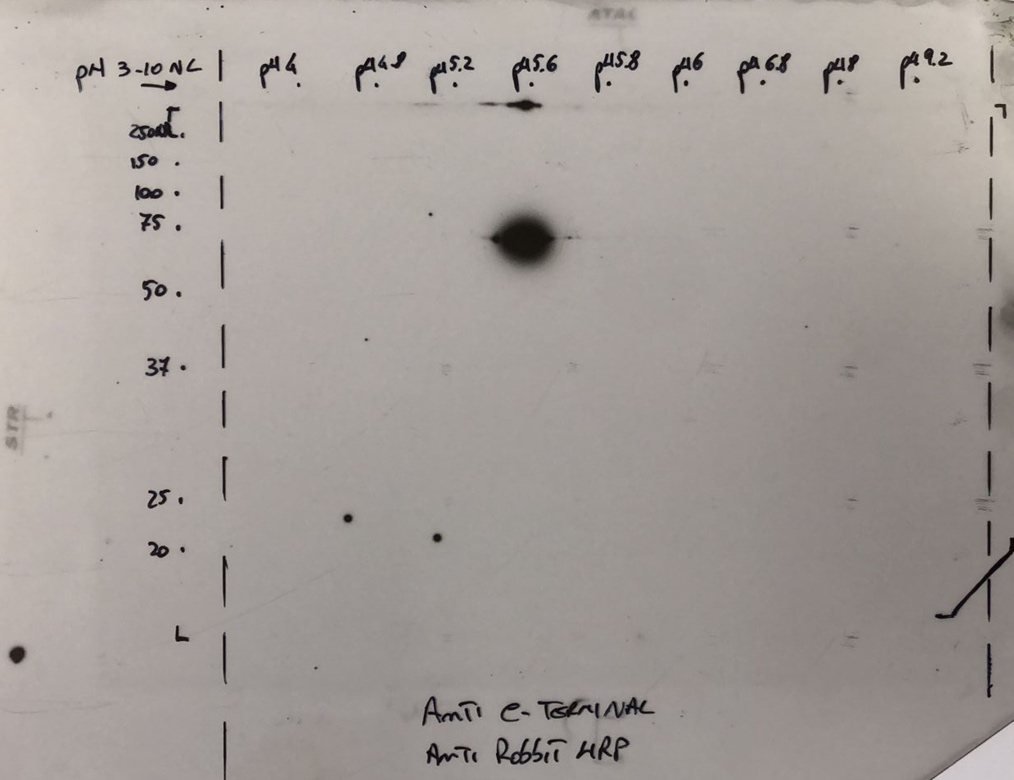
**

**
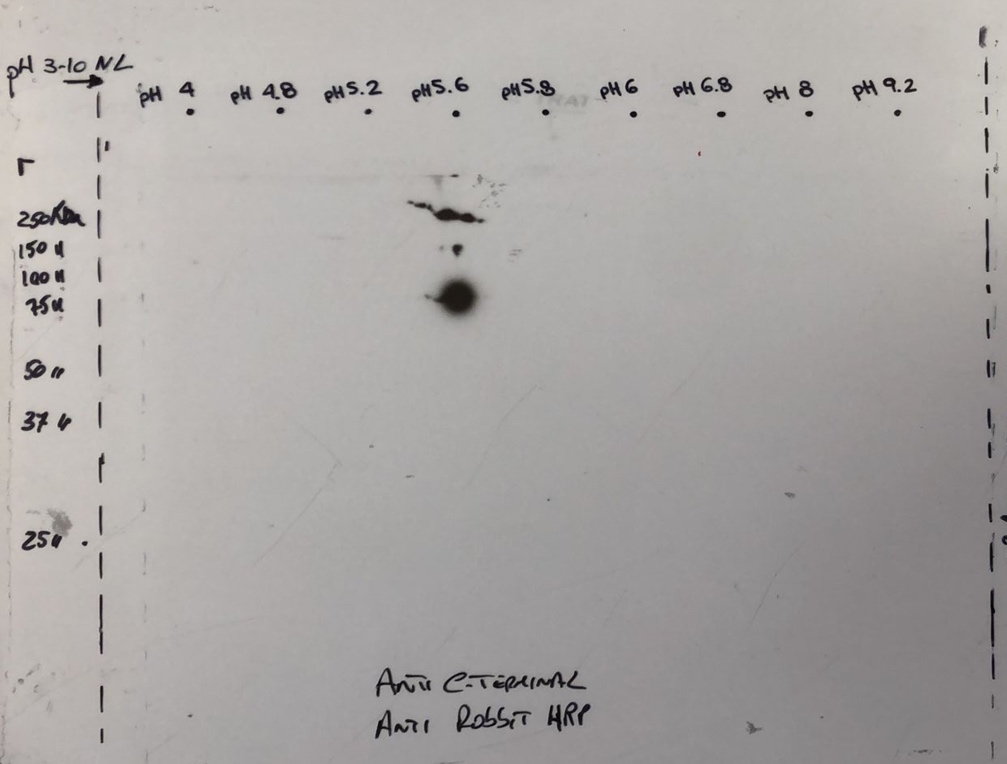
**

**Cirrhosis**

**
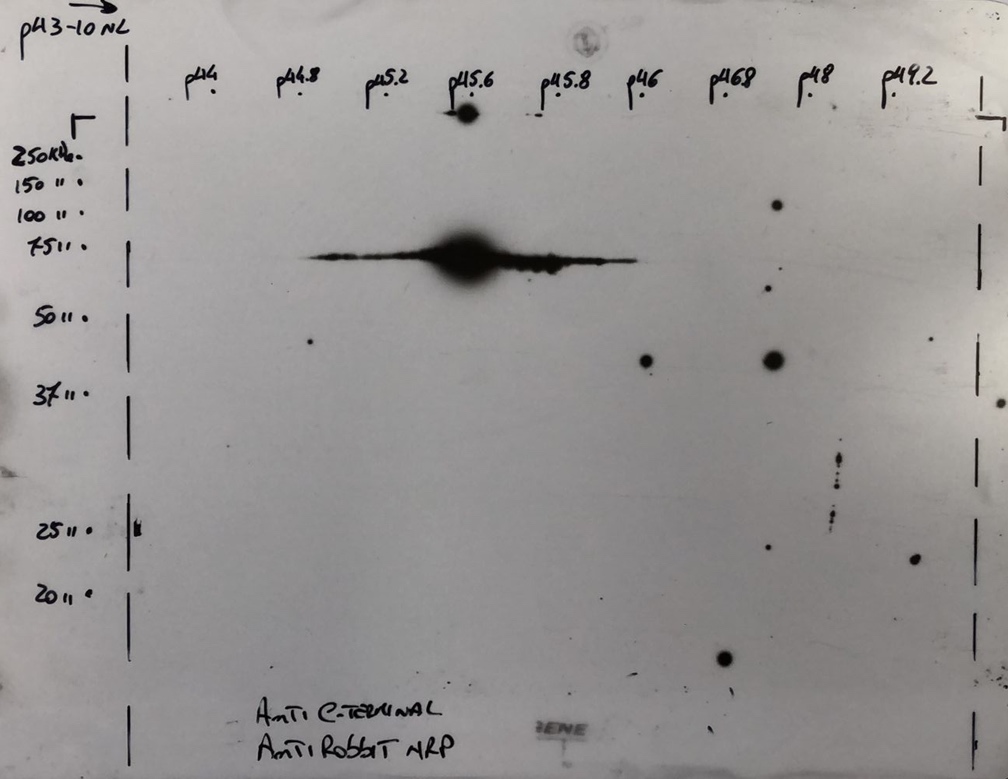
**

**
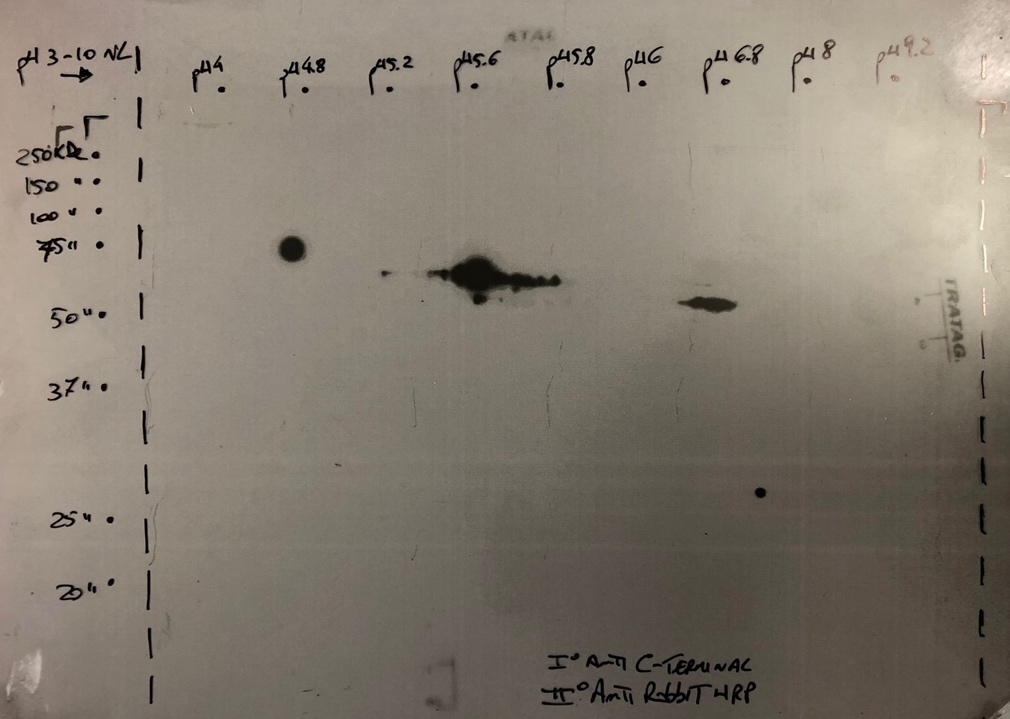
**

**HCC**

**
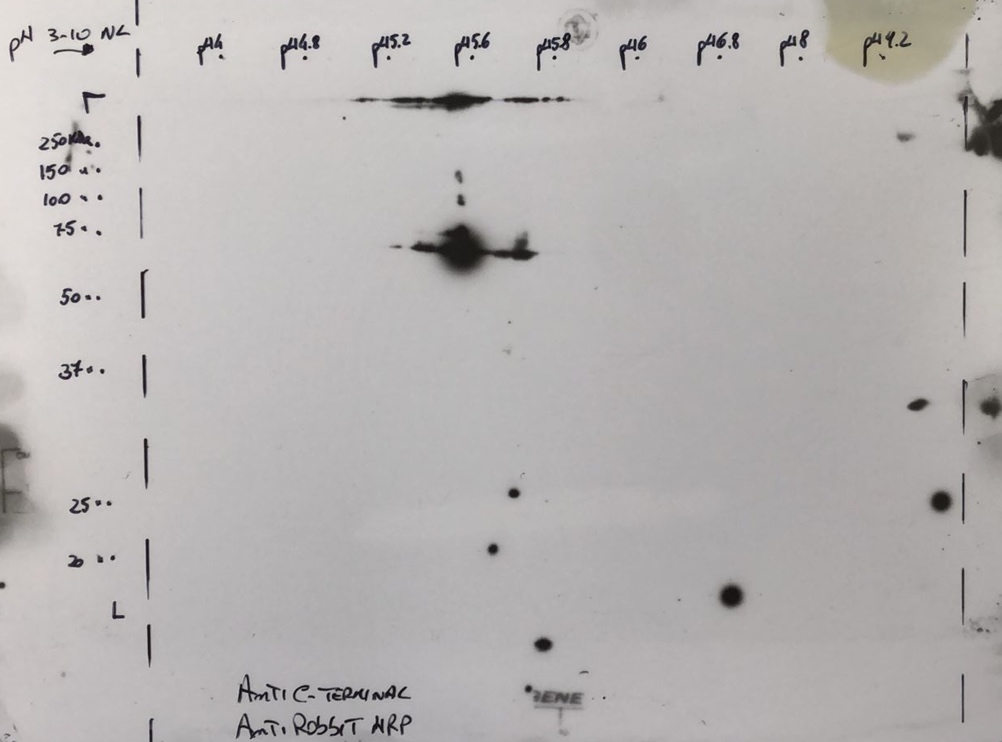
**

**
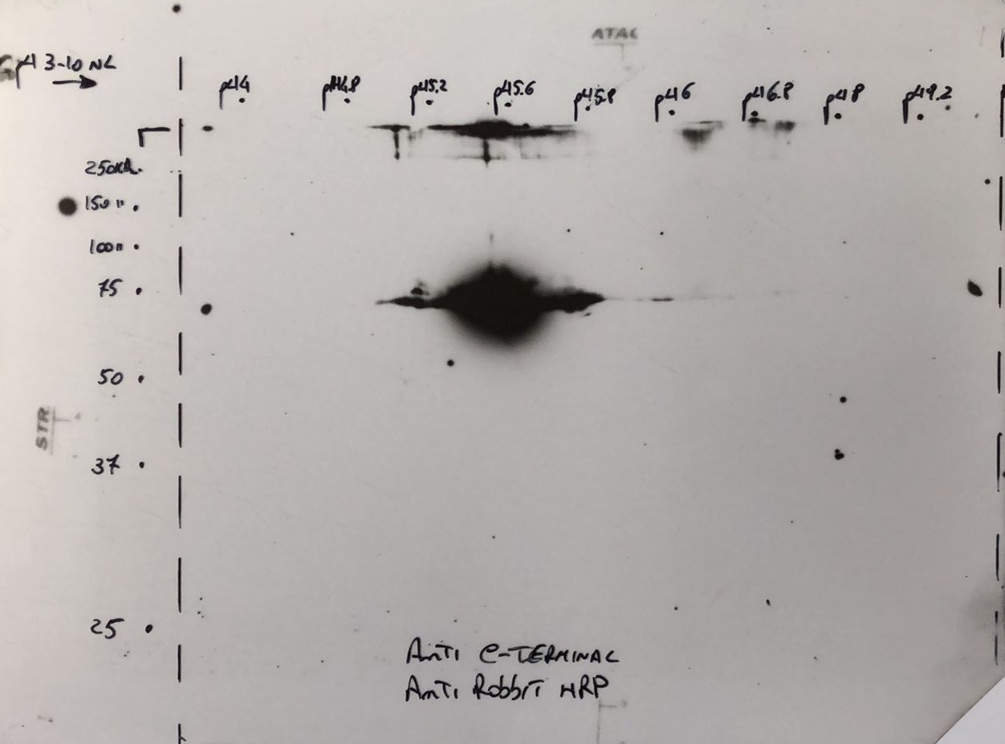
**
